# Supplementary material for: Directly synthesized cobalt oxyhydroxide as an oxygen evolution catalyst in proton exchange membrane water electrolyzers
Source: Nat Commun. 2025 Aug 13;16:7518. doi: 10.1038/s41467-025-62744-4 (PMC12350611; doi:10.1038/s41467-025-62744-4)
Supplement: Supplementary file 1 — Supplementary Information [file 41467_2025_62744_MOESM1_ESM.pdf]

Supplementary information for

Directly synthesized cobalt oxyhydroxide as an oxygen evolution catalyst in proton exchange membrane water electrolyzers

Jinzhen Huang,*[1] Zheyu Zhang,[1] Chiara Spezzati,[2] Adam H. Clark,[3] Natasha Hales,[1] Nina S. Genz,[3] Niéli Daffé,[3] Radim Skoupy,[4] Lorenz Gubler,[1] Ivano E. Castelli,[2] Thomas J. Schmidt[1,5] and Emiliana Fabbri*[1]

[1] PSI Center for Energy and Environmental Sciences, CH-5232 Villigen PSI, Switzerland

E-mail: emiliana.fabbri@psi.ch; jinzhen.huang@psi.ch

[2] Department of Energy Conversion and Storage, Technical University of Denmark, Anker Engelunds Vej 411, DK-2800, Kgs. Lyngby, Denmark

[3] PSI Center for Photon Science, CH-5232 Villigen PSI, Switzerland

[4] PSI Center for Life Sciences, CH-5232 Villigen PSI, Switzerland

[5] Institute for Molecular Physical Sciences, ETH Zürich, CH-8093 Zürich, Switzerland

Contents

Supplementary figures, notes, and tables 4

Supplementary Table 1. XRD refinement 4

Supplementary Figure 1. TEM images 5

Supplementary Table 2. Fitting of the ex-situ EXAFS spectrum 6

Supplementary Figure 2. CV analysis 7

Supplementary Figure 3. Structural characterization for Co_3O_4 control sample 8

Supplementary Figure 4. The pH-dependent Co redox process and OER activity 9

Supplementary Figure 5. Structural characterization of b-CoOOH control sample 10

Supplementary Figure 6. Electrochemical performance of b-CoOOH 11

Supplementary Figure 7. Flow cell 12

Supplementary Figure 8. CP measurement 13

Supplementary Figure 9. Operando spectra at Co K edge in 0.1 M KOH 14

Supplementary Figure 10. Shift of adsorption energy extracted by different methods 15

Supplementary Figure 11. EIS plots at different potentials 16

Supplementary Figure 12. Flat band potentials for CoOOH in pH = 1 and 13 extracted from Mott-Schottky analysis 17

Supplementary Figure 13. Operando EXAFS spectra 18

Supplementary Figure 14. Fitting of the operando EXAFS spectra 19

Supplementary Figure 15. HER/HOR of Pt in the H/D electrolytes 20

Supplementary Figure 16. CVs of CoOOH in the H/D electrolytes 21

Supplementary Note 1. Discussion on the H/D kinetic isotope effect in different electrolytes. 23

Supplementary Table 3. Discussion of O-H bond breaking for AEM 23

Supplementary Figure 17. Temperature-dependent Tafel plots 25

Supplementary Table 4. Summary of E_{app} and $\log(A_{app})$ 26

Supplementary Figure 18. DFT results of the AEM pathway 27

Supplementary Figure 19. DFT results of the OPM pathway 28

Supplementary Figure 20. CVs collected during operando measurement 29

Supplementary Figure 21. Operando XAS spectra (CV) 30

Supplementary Figure 22. Operando XAS spectra (CP) 31

Supplementary Figure 23. Fitting of the operando EXAFS spectra (CP for 1 h) 32
Supplementary Figure 24. Anode pictures of the flow cell....................................... 33
Supplementary Table 5. Co dissolution.. 34
Supplementary Figure 25. Commercial CoO_x (Sigma-Aldrich)................................ 35
Supplementary Figure 26. Activation process 36
Supplementary Figure 27. Polarization curves of 3 CCMs 37
Supplementary Figure 28. Comparison of cell potential to IrO_2/TiO_2........................ 38
Supplementary Figure 29. Onset potential 39
Supplementary Figure 30. HFR analysis.. 40
Supplementary Figure 31. Ex-situ conductivity 41
Supplementary Note 2. Cell overpotential breakdown analysis................................ 42
Supplementary Figure 32. Overpotential breakdown analysis 44
Supplementary Figure 33. Summary of overpotential breakdown analysis 45
Supplementary Note 3. Stability of CoOOH in a PEM water electrolyzer. 46
Supplementary Figure 34. HFR at 100 mA cm^{-2}.. 47
Supplementary Figure 35. Changes in overpotentials after CP at 100 mA cm^{-2} 48
Supplementary Figure 36. CP measurement at 200 mA cm^{-2} 49
Supplementary Figure 37. CP measurement at 500 mA cm^{-2} 50
Supplementary Figure 38. Changes in overpotentials after CP at 500 mA cm^{-2} 51
References in Supplementary information 52

Supplementary figures, notes, and tables

Supplementary Table 1. XRD refinement

Structural parameters determined from Rietveld refinement of the CoOOH XRD pattern.

Crystallite size (nm)	axis [0 0 1]	equatorial<br>axial	14<br>8
Microstrain	axis [0 0 1]	equatorial<br>axial	808<br>1262
Cell parameter (Å)	a<br>2.8511(6)	b<br>2.8511(6)	c<br>13.1867(2)
Atoms	Co	O	H
x	0.000	0.000	0.000
y	0.000	0.000	0.000
z	0.000	0.403	0.500
Uiso	0.067	0.067	0.010

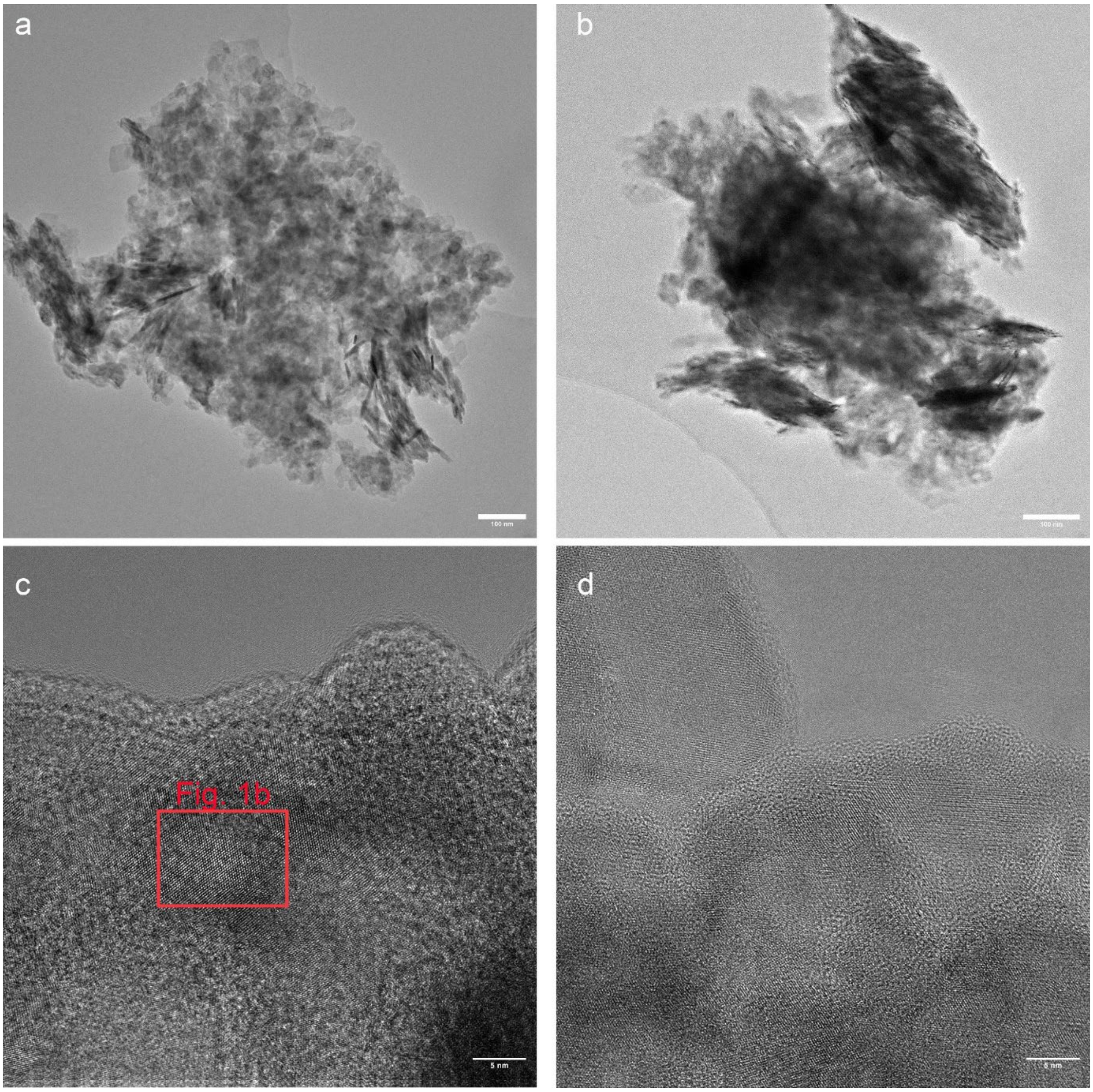

Supplementary Figure 1. TEM images

(a-d) TEM images of the as-prepared CoOOH catalysts at different magnifications. Specifically, the local region in panel c is selected and shown as Figure 1b in the main text. The scale bars in panel (a-d) are 100, 100, 5 and 5 nm, respectively.

Supplementary Table 2. Fitting of the ex-situ EXAFS spectrum

Fitting parameters of the k^3-weighted EXAFS spectrum of CoOOH in Figure 1d.

	CN	R (Å)	σ^2(Å^2)	E_o (eV)	R-factor
Co-O	6	1.903 ± 0.003	0.003 ± 0.0002	1.173	0.004
Co-Co	6	2.853 ± 0.003	0.004 ± 0.0001		

Note: The fitting was performed with a k range of 2 to 14 Å^{-1}. The coordination number (CN) for each shell was set to be 6. The amplitude reduction factor was set to be 0.80 for the fitting. E_o refers to the energy shift, R refers to Co-O bond distance, σ^2 is Debye Waller factor and the R-factor is an indicator of fit quality.

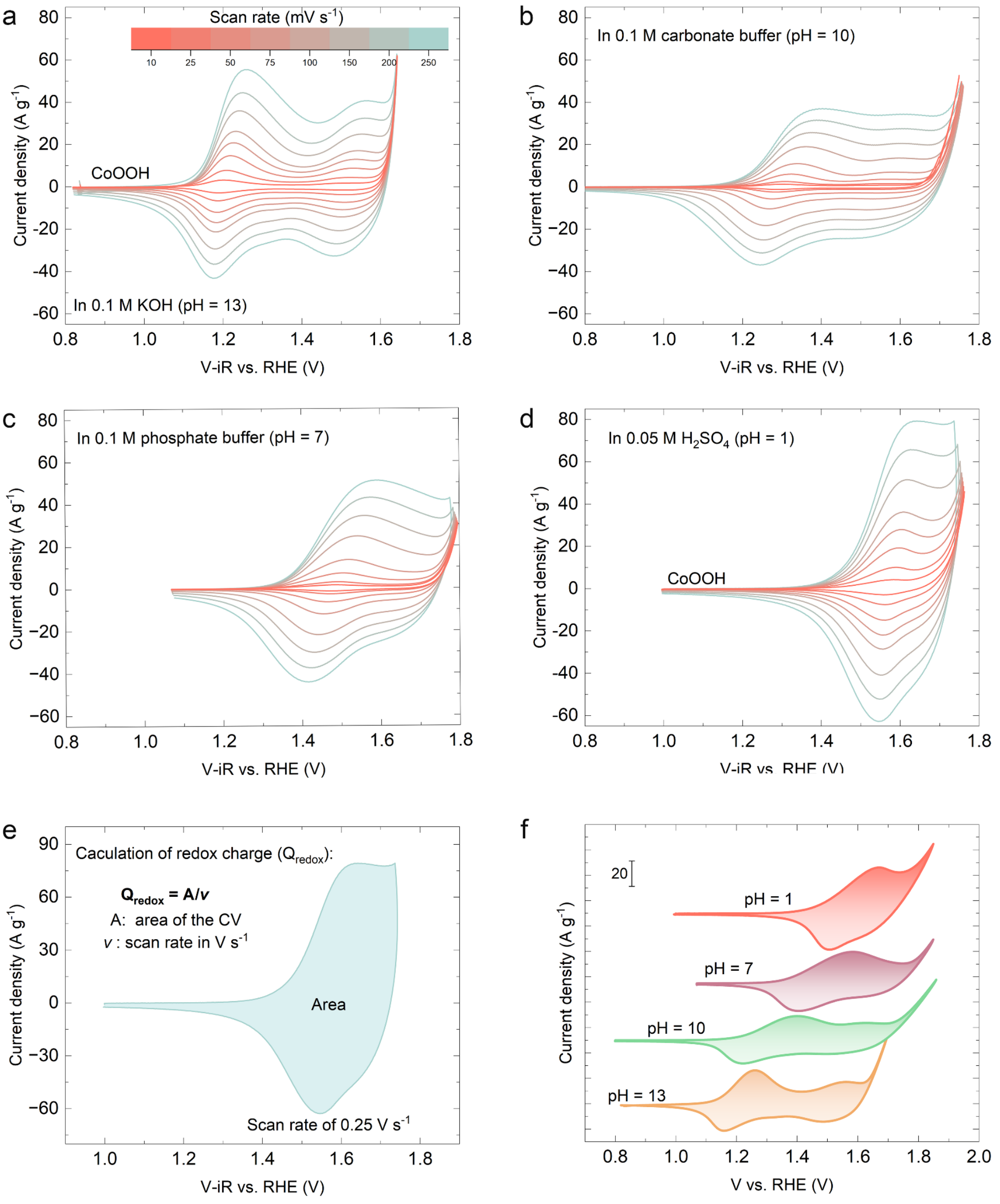

Supplementary Figure 2. CV analysis

CV analysis to understand the surface redox processes in CoOOH. The CVs have been collected at different scan rates (10 to 250 mV s^{-1}) in (a) 0.1 M KOH with pH = 13, (b) 0.1 M carbonate buffer with pH = 10, (c) 0.1 M phosphate buffer with pH = 7, and (d) in 0.05 M H_2SO_4 with pH = 1, respectively. (e) Scheme showing the calculation of redox charge (Q_{redox}) based on the CV area (Q_{redox} = *A*/*v*, where *A* is the area of the CV and *v* is the scan rate in V s^{-1}). The CV area is obtained by integration. Due to the overlap of the OER with the Co redox processes, we note that there are contributions from the charge from the OER, and also from the charge stored in the electric double layers. Therefore, the total charge calculated by the area of the CV is slightly higher than the charge passed during the interfacial redox (oxidation + reduction) processes. (f) CVs collected at the scan rate of 100 mV s-1 without *iR* correction, the iR-corrected version is shown Figure 2a. Source data are provided as a Source Data file.

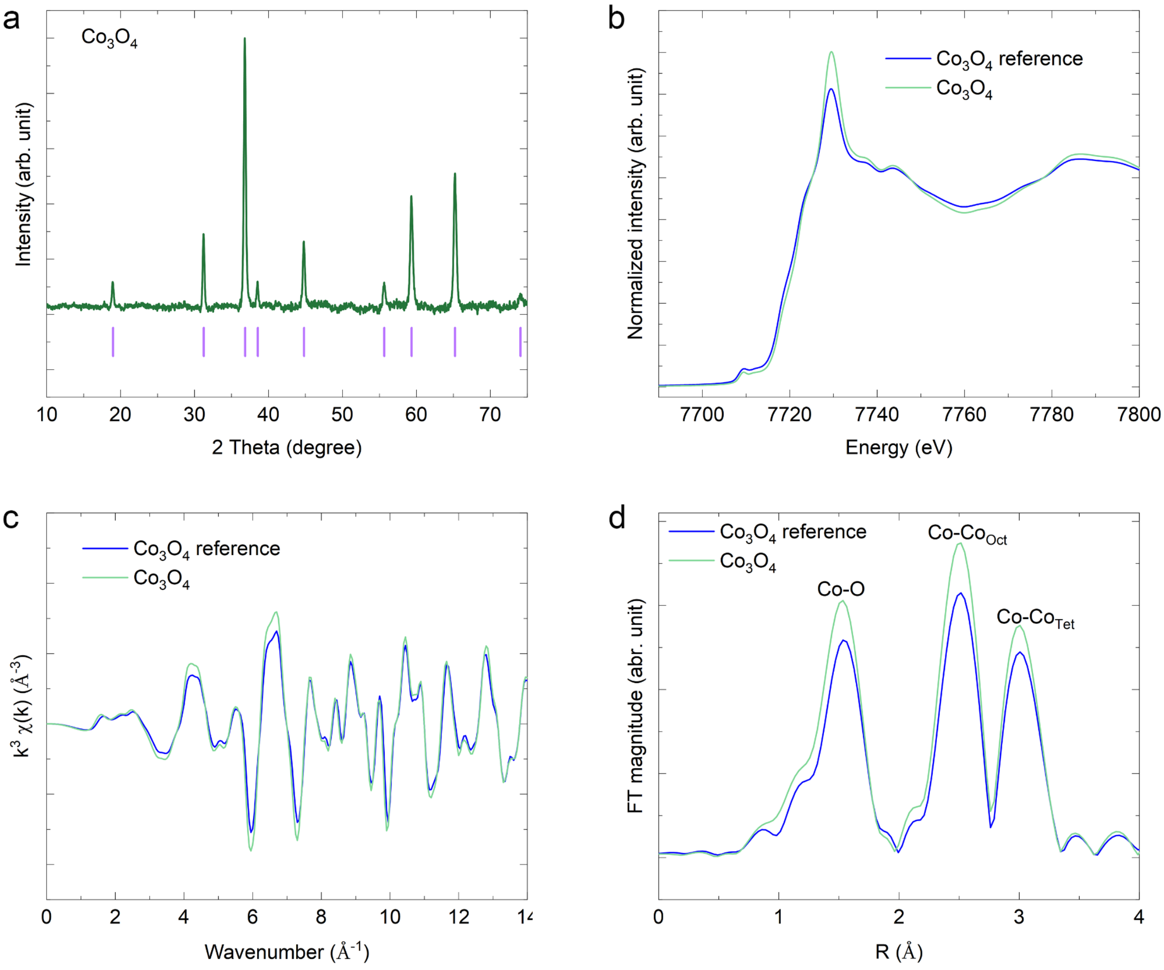

Supplementary Figure 3. Structural characterization for Co_3O_4 control sample

Structural characterizations of the synthesized Co_3O_4 control sample. The precursor of this Co_3O_4 control sample is synthesized by following a similar precipitation process as for the CoOOH catalyst, before being dried overnight and annealed in air at 500 °C for 2h to get the spinel Co_3O_4 structure (See Methods for more details). (a) the XRD pattern matches well with the standard pattern of a spinel structure. (b) The XANES at the Co K edge for the as-prepared Co_3O_4 control sample, in comparison to a bulk Co_3O_4 reference sample that is commercially available from Sigma-Aldrich. (c) The k^3-weighted EXAFS spectra. (d) The corresponding FT EXAFS spectra. Source data are provided as a Source Data file.

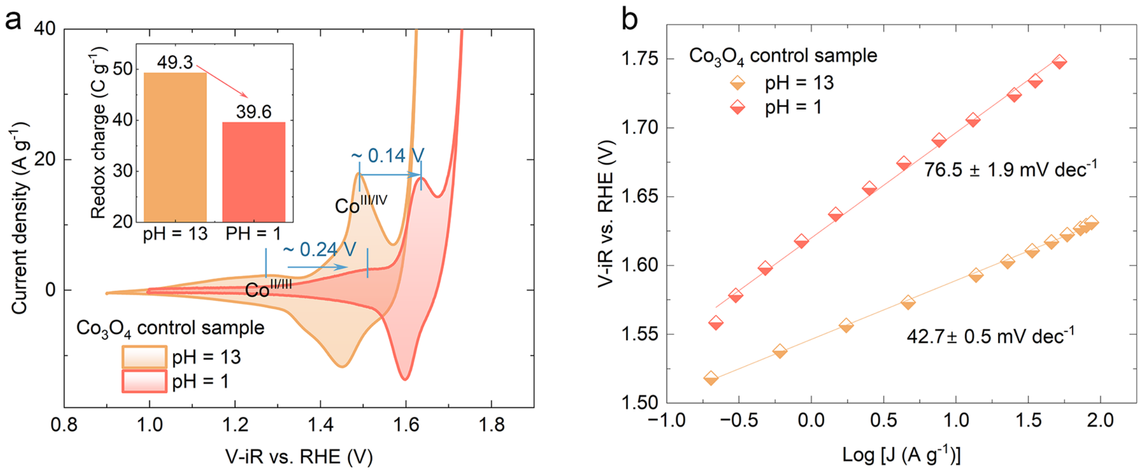

Supplementary Figure 4. The pH-dependent Co redox process and OER activity

Comparison of (a) CV curves and (b) Tafel plots in the electrolytes of pH = 13 and 1. The inset in (a) shows the redox charges (Q_{redox}) extracted by integrating the area of the corresponding CV curves (Q_{redox} = Area/*v*, where *v* is the scan rate in V s^{-1}). Source data are provided as a Source Data file.

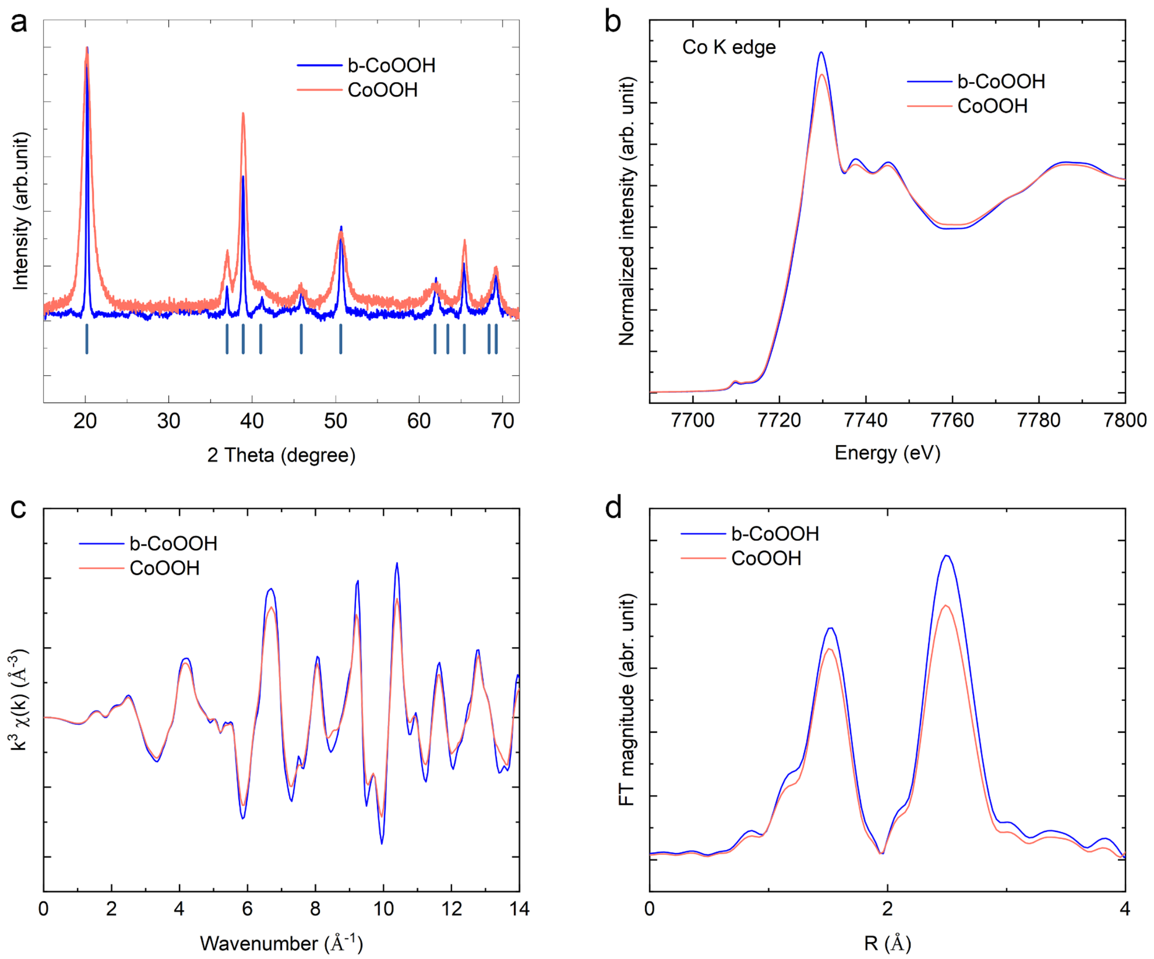

Supplementary Figure 5. Structural characterization of b-CoOOH control sample

Structural characterizations of a b-CoOOH control sample. This control sample also has a CoOOH structure, but is synthesized from a beta-$Co(OH)_2$ precursor (also used as the reference sample in Figure 1c) which is commercially available from Sigma Aldrich (See Methods for more details). Therefore, it is denoted as b-CoOOH to be differentiated from the more interesting CoOOH sample. (a) Comparison of the XRD patterns between b-CoOOH and CoOOH. The full width at half maximum (FWHM) of the XRD pattern for b-CoOOH control sample is smaller than that of the CoOOH sample (Figure 1b), indicating b-CoOOH has better crystallinity. (b) The X-ray adsorption near-edge structure (XANES) at the Co K edge for the b-CoOOH control sample, in comparison to the CoOOH sample. (c) The k^3-weighted extended X-ray absorption fine structure (EXAFS) spectra. (d) The corresponding Fourier-transformed (FT) EXAFS spectra. Source data are provided as a Source Data file.

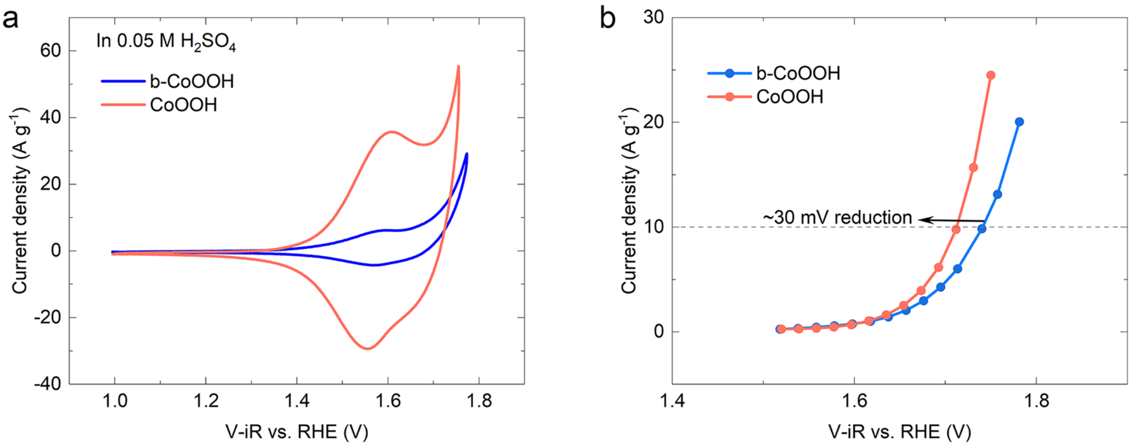

Supplementary Figure 6. Electrochemical performance of b-CoOOH

Comparison of (a) the cyclic voltammograms (CVs) and (b) OER polarization curves of the b-CoOOH control sample and the CoOOH sample in 0.05 M H_2SO_4. The CVs were collected at a scan rate of 100 mV s^{-1}. Both samples show only one redox pair in an acidic environment. To avoid the influence from the redox current, the OER polarization curves were collected using the steady state chronoamperometry (CA) technique. A reduction of ~30 mV in the overpotential at the current density of 10 A g^{-1} is observed in CoOOH when compared to b-CoOOH, consistent with general understanding on the relationship between catalytic activity and crystallinity. Source data are provided as a Source Data file.

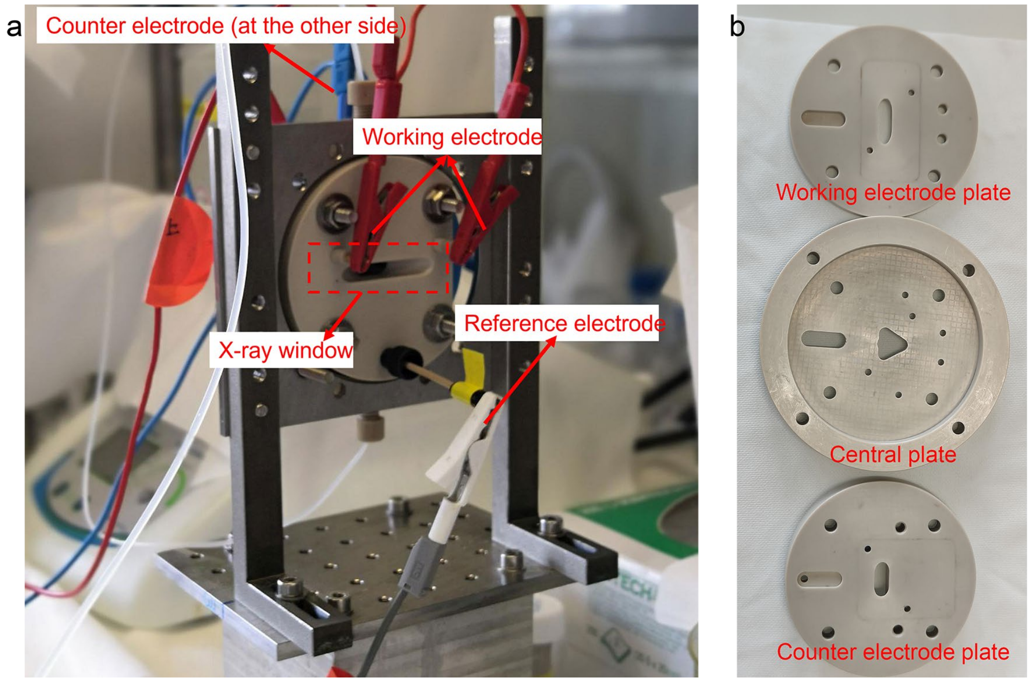

Supplementary Figure 7. Flow cell

Photos of (a) the flow cell used for operando XAS characterizations and (b) the main PEEK cell components. The schematic diagram and other details about this operando flow cell can be found in previous publications[1, 2].

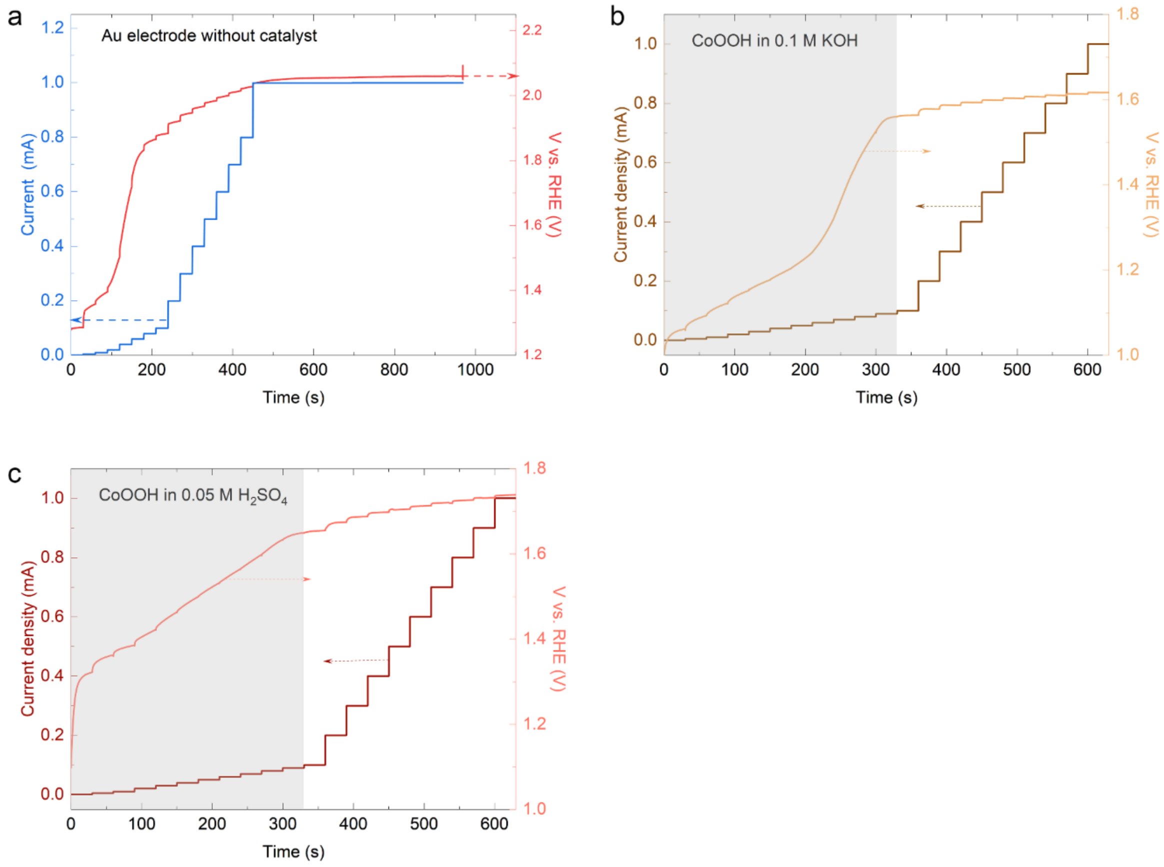

Supplementary Figure 8. CP measurement

The chronopotentiometry (CP) measurement during operando XAS characterizations. All operando measurements are performed in the flow cell depicted in Supplementary Figure 7. The control current (mA, without normalization to the mass of the catalyst) was kept constant for 30s during each step and the potential of the electrode (catalyst on Au-coated Kapton foil) was recorded. The potential and current are plotted as a function of the time for (a) the Au electrode without the catalyst, (b) CoOOH in 0.1 M KOH, and (c) CoOOH in 0.05 M H_2SO_4. The resistances (*R*) for CoOOH are 10.3 and 5.6 Ω in 0.1 M KOH and 0.05 M H_2SO_4, respectively. Source data are provided as a Source Data file.

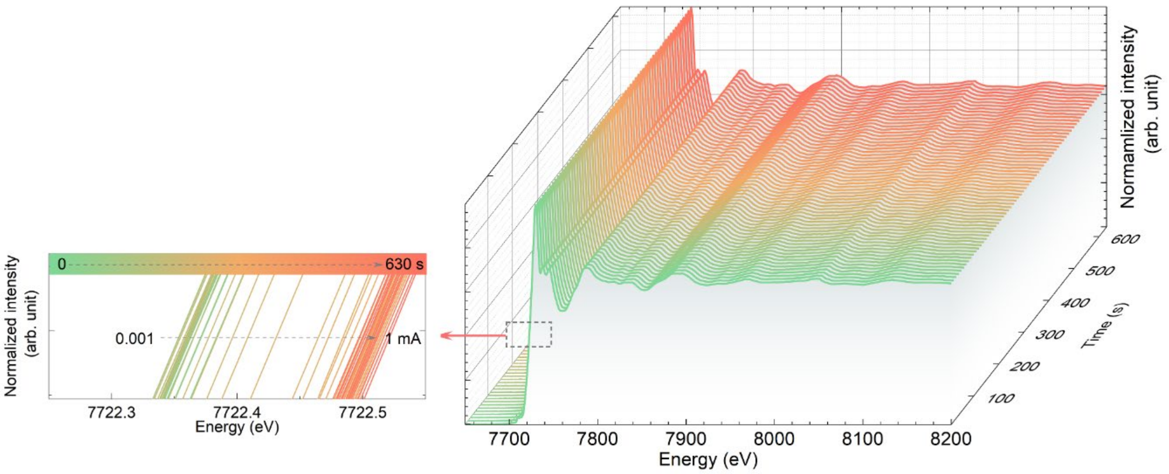

Supplementary Figure 9. Operando spectra at Co K edge in 0.1 M KOH

The operando spectra at the Co K edge for CoOOH during the CP measurements in 0.1 M KOH. There is a distinct shift in the Co edge position when increasing the control current. Source data are provided as a Source Data file.

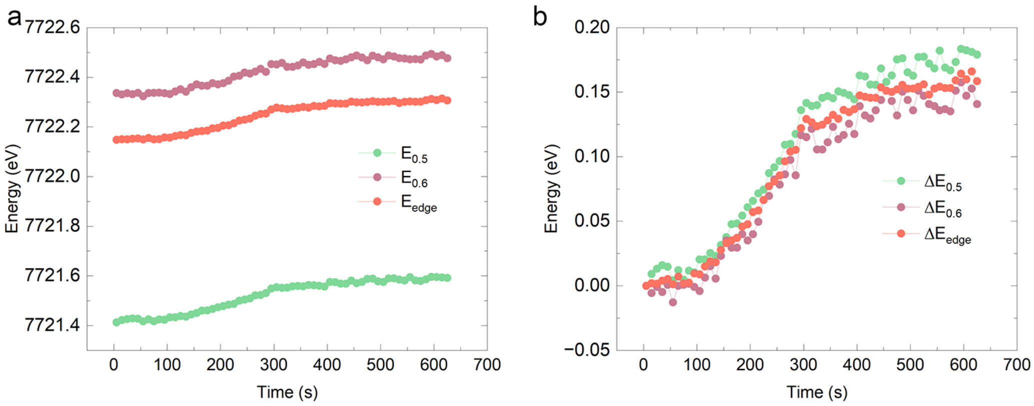

Supplementary Figure 10. Shift of adsorption energy extracted by different methods

(a) E_{edge} determined from the integral method are compared to the energies at μ = 0.5 (i.e., at half of the edge-jump intensity) and μ = 0.6 of the normalized intensity, which are denoted as $E_{0.5}$ and $E_{0.6}$, respectively. (b) Comparison of the shifts of adsorption energy extracted by different methods, i.e., ΔE_{edge}, $\Delta E_{0.5}$ and $\Delta E_{0.6}$, made by subtracting the E_{edge} at 0 s. Source data are provided as a Source Data file.

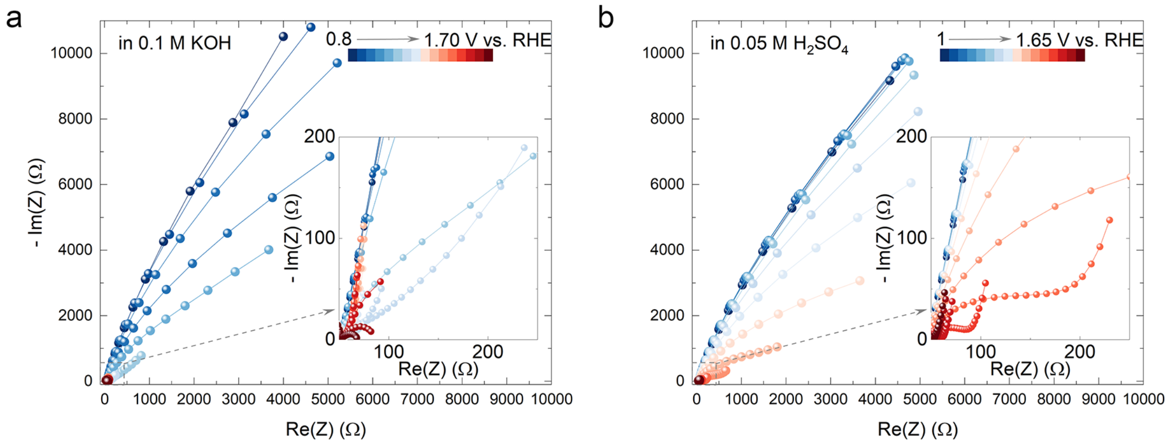

Supplementary Figure 11. EIS plots at different potentials

EIS characterization to extract the flat band potentials for CoOOH (a) in 0.1 M KOH with pH = 13 and (b) in 0.05 M H_2SO_4 with pH = 1. The insets in (a-b) show the magnification in the lower Re(Z)/-Im(Z) regions. Source data are provided as a Source Data file.

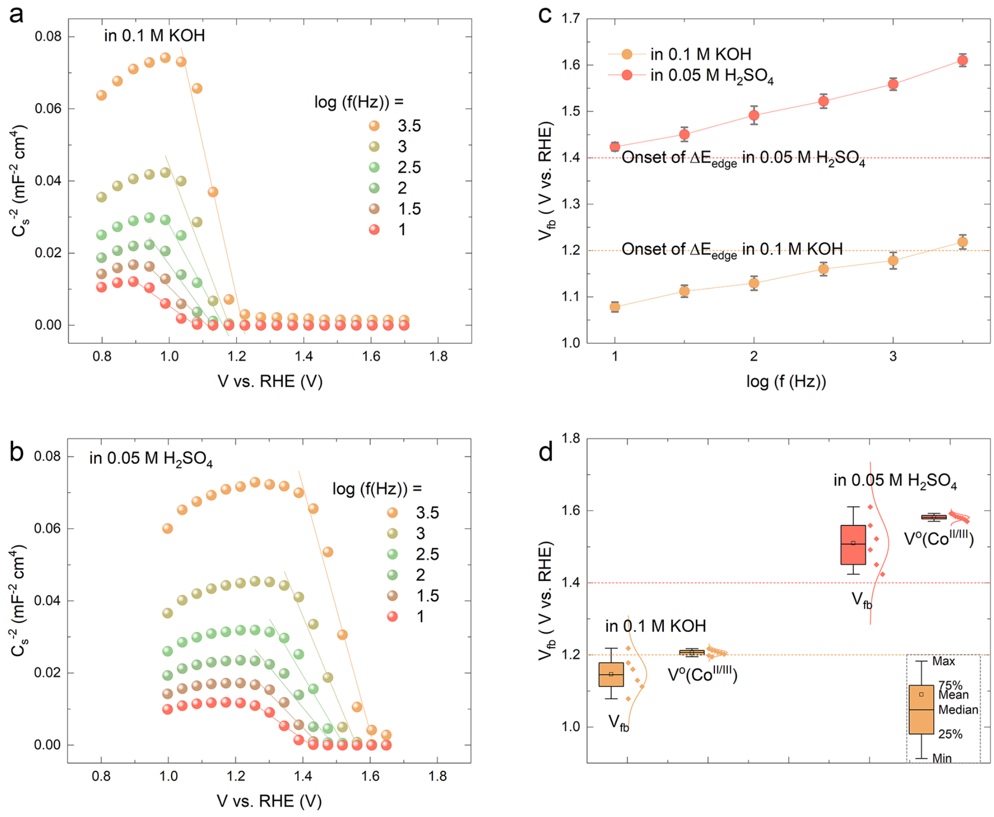

Supplementary Figure 12. Flat band potentials for CoOOH in pH = 1 and 13 extracted from Mott-Schottky analysis

Mott-Schottky plots used to extract flat band potential (V_{fb}) for CoOOH (a) in 0.1 M KOH with pH = 13 and (b) in 0.05 M H_2SO_4 with pH = 1, at different frequencies from the EIS characterizations. (c) The extracted V_{fb} are plotted as a function of log(f(Hz)) in alkaline and acidic electrolytes. (d) Comparison on the V_{fb} and $V^o(Co^{II/III})$, in alkaline and acidic electrolytes, respectively. The horizontal dashed lines in (c-d) are the onset potentials for ΔE_{edge} determined from operando hXAS characterizations. The coloured lines beside the box in (d) show the distribution of the data points. Source data are provided as a Source Data file.

Discussion: The electrochemical impedance spectroscopy data (Supplementary Figure 11) were collected with a Staircase Potentio Electrochemical Impedance Spectroscopy (Mott-Schottky) technique using the Biologic software. The frequency was changed from 100 kHz to 1 Hz. The potential windows for the measurement in 0.05 M H_2SO_4 and 0.1 M KOH are 1.0 to 1.65 V vs. RHE and 0.8 to 1.7 V vs. RHE, respectively. The $1/C_s^2$ at different frequencies can directly be extracted using this software and were plotted against the applied potentials to derive Mott-Schottky plots[3] in Supplementary Figure 12a-b. The flat band potential is determined from the x-intercept of the linear fit, as shown in Supplementary Figure 12c.

For Co-based catalysts, the V_{fb} determined by Mott-Schottky analysis is frequency dependent and is usually located at around the Co redox potentials. More

importantly, the onset of the ΔE_{edge} is consistently located close to the V_{fb} (ref.[4, 5, 6]). Here, we also show that the onset of the ΔE_{edge} is close to the V_{fb} for CoOOH in both alkaline and acidic electrolytes. The consistent observations could be due to the fact that these three parameters, namely, the onset of ΔE_{edge}, V_{fb}, and $V^{o}(Co^{II/III})$ are closely correlated with the interfacial Co oxidation in Co-based catalysts. However, slight deviations in these parameters and their pH-dependence could result from limitations of the different techniques used to extract these parameters, as well as the varied interfacial properties in different electrocatalytic systems.

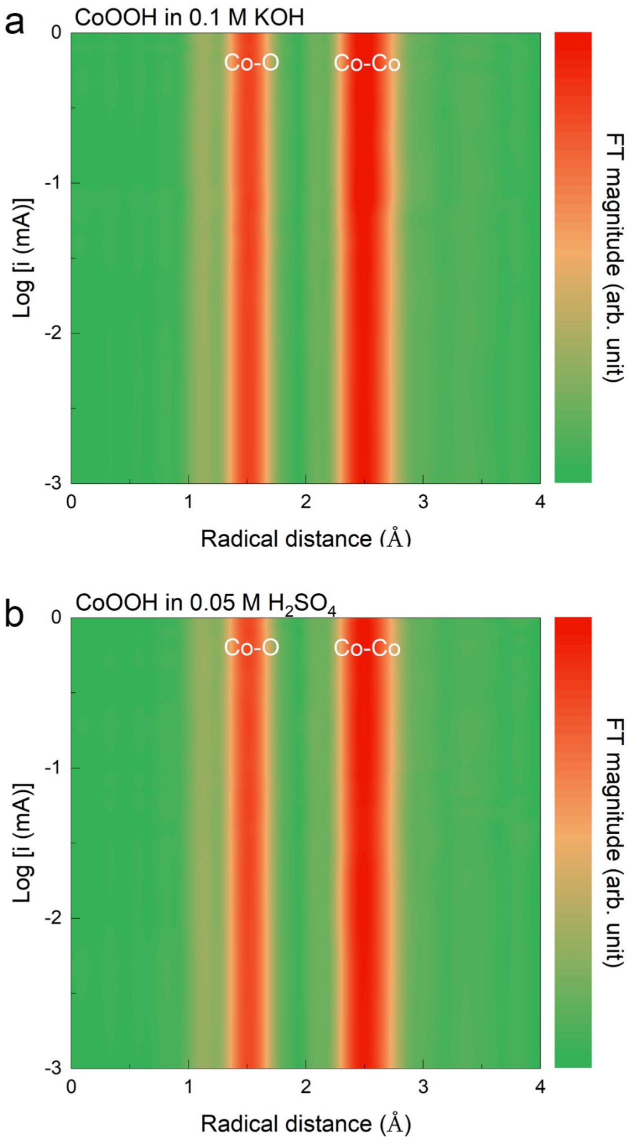

Supplementary Figure 13. Operando EXAFS spectra

The k^3-weighted Fourier-transformed (FT) EXAFS spectra for (a) CoOOH in 0.1 M KOH, (b) CoOOH in 0.05 M H_2SO_4. The y-axis is the logarithm of the reaction current during operando characterizations. Source data are provided as a Source Data file.

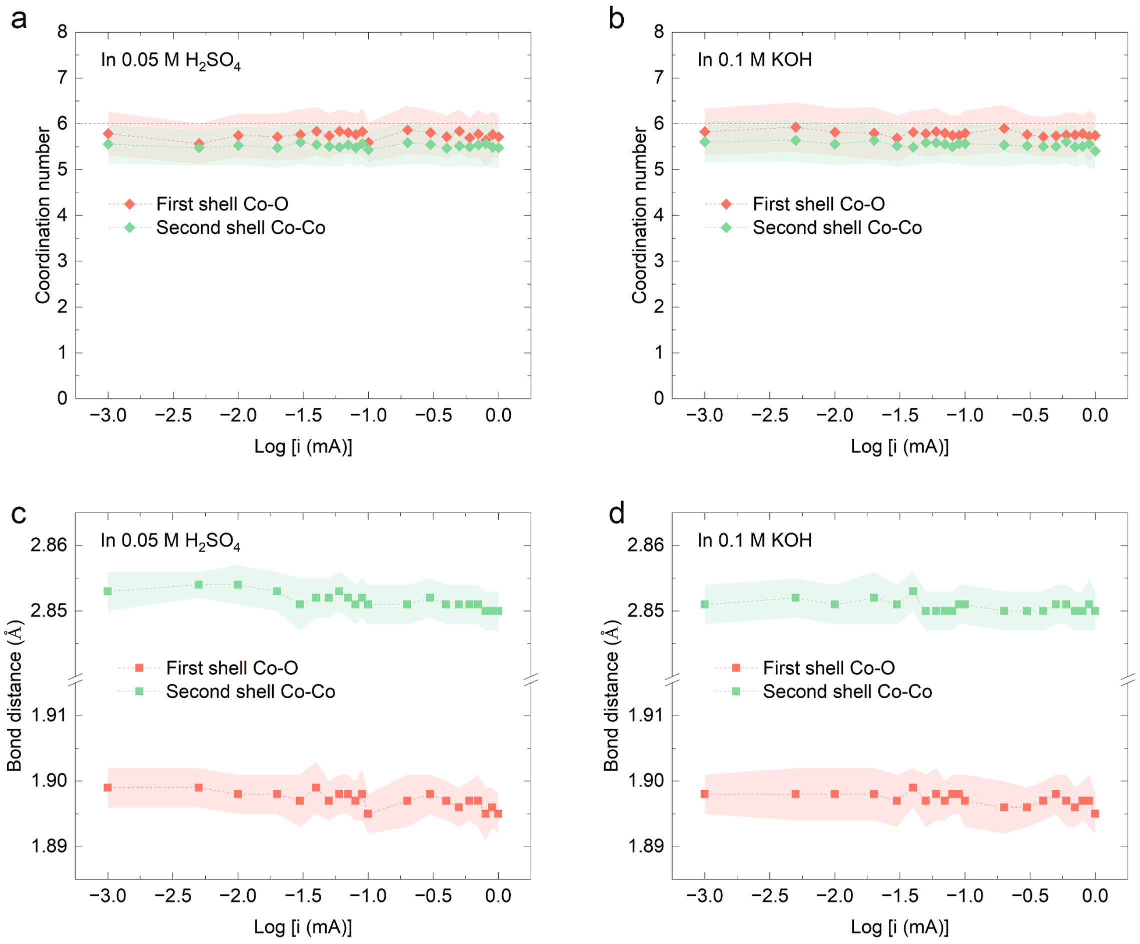

Supplementary Figure 14. Fitting of the operando EXAFS spectra

(a-b) The coordination number (CN) and (c-d) the bond distance for the Co-O shell and Co-Co shell extracted from fitting the k^3-weighted EXAFS spectra in Supplementary Figure 13, plotted as a function of log[i(mA)]. The error band represents the error from fitting the spectra in Artemis software. The fitting was performed with a k range of 2 to 14. The coordination number (CN) was obtained by assuming that the amplitude reduction factor is 0.80 for the fitting. The energy shift (E_o) for fitting falls in the range of 0.5 to 1.5 eV, the Debye Waller factor (σ^2) falls in the range of ~0.002 to 0.004. The R-factor, an indicator of fit quality, falls in the range of ~0.004 to 0.007. Source data are provided as a Source Data file.

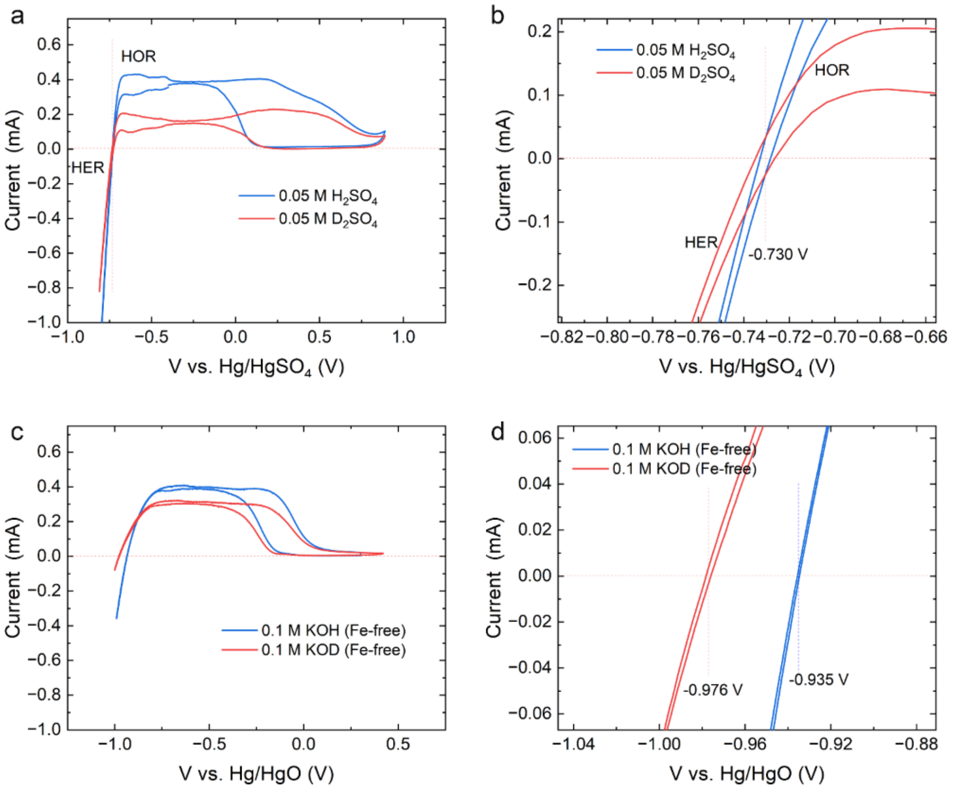

Supplementary Figure 15. HER/HOR of Pt in the H/D electrolytes

The hydrogen evolution/oxidation reaction (HER/HOR) curves are recorded to determine the potential difference between reversible hydrogen/deuterium electrode (RHE/RDE) and Hg/HgO (or $Hg/HgSO_4$) electrode. The measurement was performed by using a polycrystalline Pt disk (0.5 mm in diameter) as the working electrode, and another Pt mesh as the counter electrode. The electrolytes were saturated with H_2 before the measurement. CV curves for (a-b) acidic and (c-d) alkaline electrolytes, respectively, were collected at a scan rate of 20 mV s^{-1} and at a rotation speed of 1600 rpm. Panel b (or d) is the enlarged figure of panel a (or c), to highlight intercept of the CV curves. The potential difference is determined from the average of the x-intercepts of the cathodic and anodic scans. Source data are provided as a Source Data file.

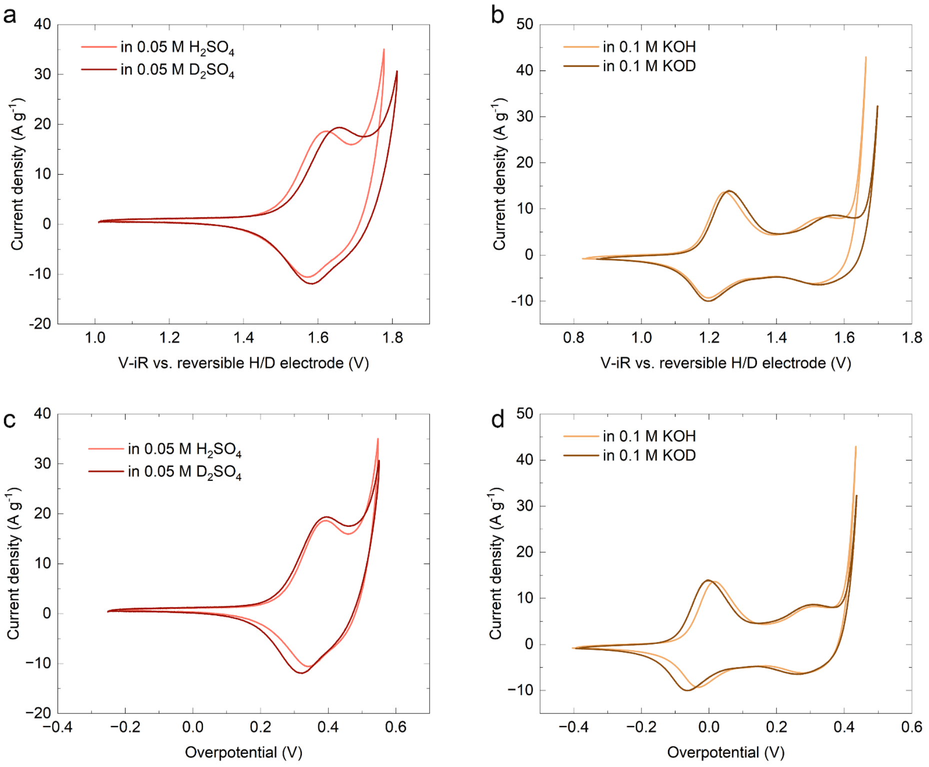

Supplementary Figure 16. CVs of CoOOH in the H/D electrolytes

Comparison of CV curves on different potential scales. (a-b) CV curves of CoOOH on the RHE/RDE scale in the (a) acidic electrolyte and (b) alkaline electrolyte. (c-d) CV curves of CoOOH on the overpotential scale in the (c) acidic electrolyte and (d) alkaline electrolyte. The resistances for iR-correction are 62 ± 5, 90 ± 1, 59 ± 3, 86.6 ± 0.3 for 0.1 M KOH, 0.1 M KOD, 0.05 M H_2SO_4, 0.05 M D_2SO_4, respectively. Error bars represent the standard deviations of three replicates. Source data are provided as a Source Data file.

The CV curves of CoOOH recorded in alkaline and acidic electrolytes, with the potential calibrated on the RHE/RDE scale are present at Supplementary Figure 16a-b. Both the Co redox peaks and the onset of the OER are anodically shifted after D substitution in the electrolyte. We note that the equilibrium potential for the OER is shifted from 1.229 V vs. RHE to 1.262 V vs. RDE after D substitution[7, 8], which contributes to the observed anodic shift on the RHE/RDE scale. To obtain the CV curves on an overpotential scale, the conversion in the H-electrolyte is shown in the equation S4:

$$\text{Overpotenital} = E(\text{RHE}) - 1.229\ \text{V} \qquad \text{(S4)}$$

The conversion in the D-electrolyte should follow equation S5:

$$\text{Overpotenital} = E(\text{RHE}) - 1.262\ \text{V} \qquad \text{(S5)}$$

Finally, CV curves of CoOOH on the overpotential scale are shown in Supplementary Figure 16c-d. Clearly, the onset of the OER is very similar after D substitution, no matter in alkaline or acidic electrolytes. The data shown in Figure 3c-d is treated following the same procedure outlined above.

Supplementary Note 1. Discussion on the H/D kinetic isotope effect in different electrolytes.

The reactant is different when the pH of the electrolyte is shifted from alkaline to acidic electrolytes. In a conventional 4-electron OER process, the formula for each step is shown in Supplementary Table 2. Specifically, the break of an O-H bond is taken as an important indicator to evaluate the existence of the KIE in each step.

Supplementary Table 3. Discussion of O-H bond breaking for AEM

Summary of different OER steps in a conventional absorbate evolution mechanism (AEM) in alkaline and acidic electrolytes, with OH^- and H_2O as reactants, respectively.

Electrolyte	Steps	Break of O-H bond
**In alkaline electrolyte**	I. * + **OH^-** → *OH + e^-	No
	II. *OH + **OH^-** → *O + H_2O + e^-	Yes
	III. *O + **OH^-** → *OOH + e^-	No
	IV. *OOH + **OH^-** → O_2(gas) + H_2O + e^-	Yes
**In acidic electrolyte**	I. * + **H_2O** → *OH + H^+ + e^-	Yes
	II. *OH → *O + H^+ + e^-	Yes
	III. *O + **H_2O** → *OOH + H^+ + e^-	Yes
	IV. *OOH → O_2(gas) + + H^+ + e^-	Yes

According to quantum mechanics, the zero-point energy (ε_0) of a molecule is related to the ground state vibration energy[9]:

$$\varepsilon_0 = \frac{1}{2}\mathrm{h}\nu \tag{S6}$$

where h is Plank's constant, and ν is the vibration frequency:

$$\nu = \frac{1}{2\pi}\sqrt{\frac{\mathrm{k}}{m}} \tag{S7}$$

where k is the force constant of a bond, and m is the reduced mass of two atoms in a bond. In the O-H bond, the reduced mass

$$m_{O-H} = \frac{m_O * m_H}{m_O + m_H} \tag{S8}$$

Where m_O and m_H are the mass of O and H respectively. Since the mass of D is twice of H, then a primary KIE is observed when changing from H to D, as this results in different zero-point energies of O-H and O-D bonds[10]. Specifically,

$$\frac{\varepsilon_{0,O-D}}{\varepsilon_{0,O-H}} = \frac{\nu_{O-D}}{\nu_{O-H}} \sim \sqrt{\frac{m_{O-H}}{m_{O-D}}} = \sqrt{\frac{m_H}{m_D}} * \sqrt{\frac{(m_H + m_O)}{(m_D + m_O)}} \sim 0.687 \tag{S9}$$

Theoretically, a step involving the break of an O-H bond experiences a significant change in the zero-point energy, and thus the reaction rate.

Furthermore, in alkaline electrolytes, the change in zero-point energy for the formation of *OOH (step III in Supplementary Table 3) after substitution of H by D can be estimated as the following:

$$\frac{\varepsilon_{0,O-OD}}{\varepsilon_{0,O-OH}} = \frac{\nu_{O-OD}}{\nu_{O-OH}} \sim \sqrt{\frac{m_{O-OH}}{m_{O-OD}}} = \sqrt{\frac{m_{OH}}{m_{OD}}} * \sqrt{\frac{(m_{OH} + m_O)}{(m_{OD} + m_O)}} \sim 0.957 \tag{S10}$$

Therefore, the change in zero-point energy in the formation of *OOH is relatively small when compared to steps involving a break in an O-H bond (e.g., water dissociation and deprotonation).

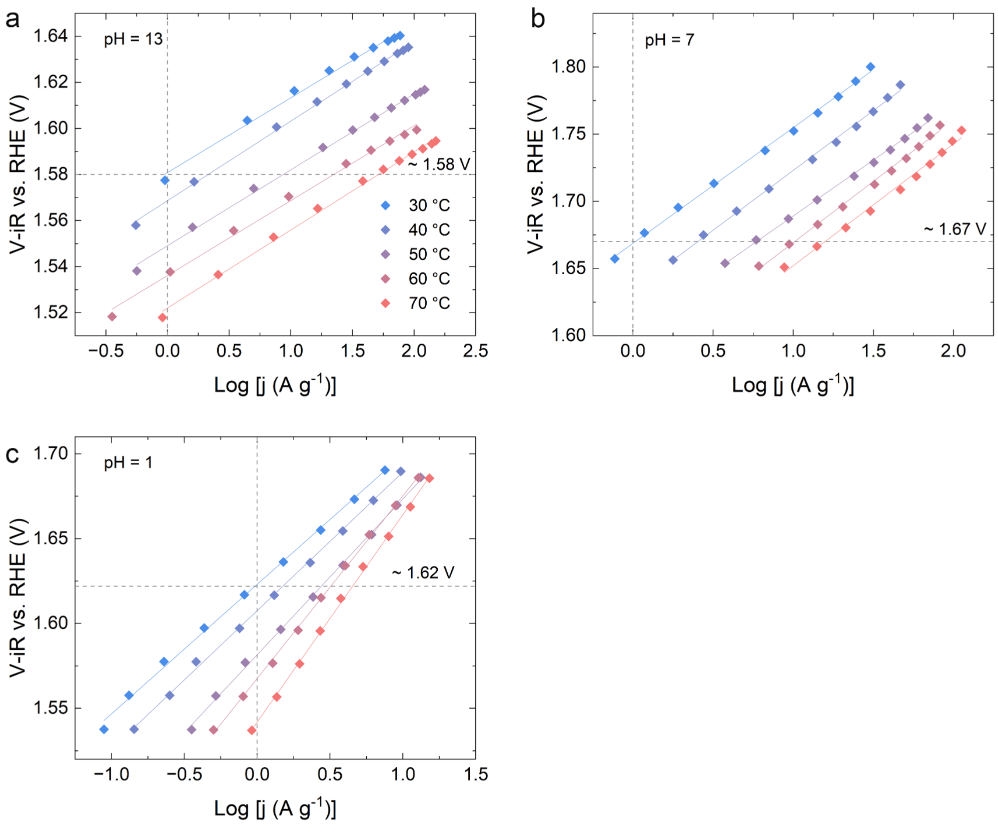

Supplementary Figure 17. Temperature-dependent Tafel plots

(a-c) Tafel plots collected at different temperatures (30, 40, 50, 60, and 70 °C) and in (a) alkaline, (b) neutral and (c) acidic electrolytes, respectively. The horizontal dashed curve marks the onset potential, i.e., the applied potential at log(j(A g^{-1})) = 0 in each electrolyte. The Arrhenius plots with log (j(A g^{-1})) at the onset potential is plotted as a function of 1000/T in Figure 4b, to extract the E_{app} and log(A_{app}) in the inset of Figure 4b and Supplementary Table 4 below. Source data are provided as a Source Data file.

Supplementary Table 4. Summary of E_{app} and $\log(A_{app})$

Summary of E_{app} and $\log(A_{app})$ derived from **Supplementary Figure 17d** for different electrolytes.

	Intercept/$\log(A_{app})$[a]	Slope	E_{app}(kJ mol^{-1})[b]	E_{app}(eV)[c]
pH = 13	15.35 ± 0.72	4.66 ± 0.23	89.31 ± 4.42	0.93 ± 0.05
pH = 7	10.24 ± 0.65	3.09 ± 0.21	59.15 ± 4.01	0.61 ± 0.04
pH = 1	5.68 ± 0.48	1.72 ± 0.15	32.90 ± 2.97	0.34 ± 0.03

Note:

a. The $\log(A_{app})$ is obtained from the intercept of the fit in Supplementary Figure 17d.

b. Then E_{app} (in kJ mol^{-1}) is calculated from the slope by E_{app} = -slope* 2.303.

c. 1 kJ mol^{-1} = 0.01036410 eV.

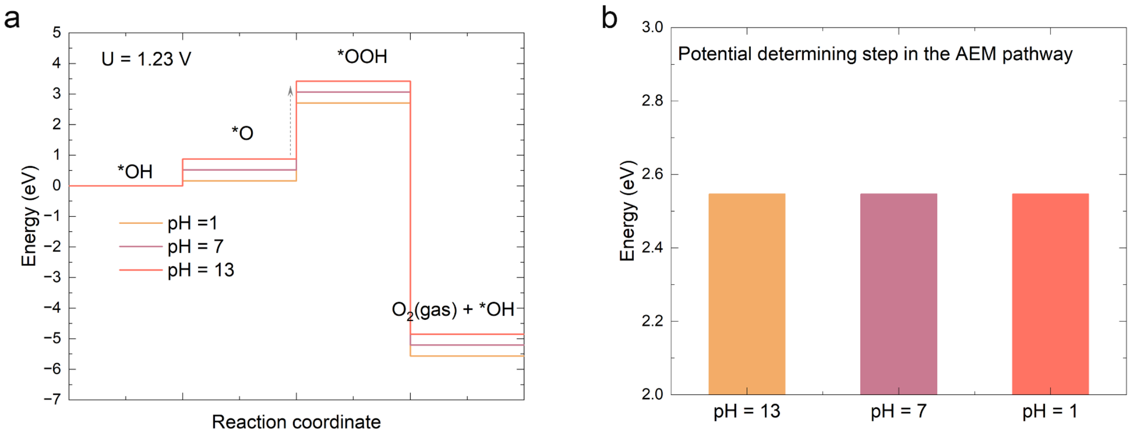

Supplementary Figure 18. DFT results of the AEM pathway

Density function theory (DFT) calculations on the (10-14) surface of CoOOH, which has been reported to be most stable, with a coverage of 1 mL H_2O which dissociates immediately into *OH (ref.[11]). The energy for each step was corrected to the potential of 1.23 V. More details are provided in Methods. (a) The energy diagram for the AEM in different pH environments. The potential determining step (PDS) is the formation of *OOH, as indicated by a dashed arrow. (b) The energies for the PDS of the AEM are compared, and are pH independent. Source data are provided as a Source Data file.

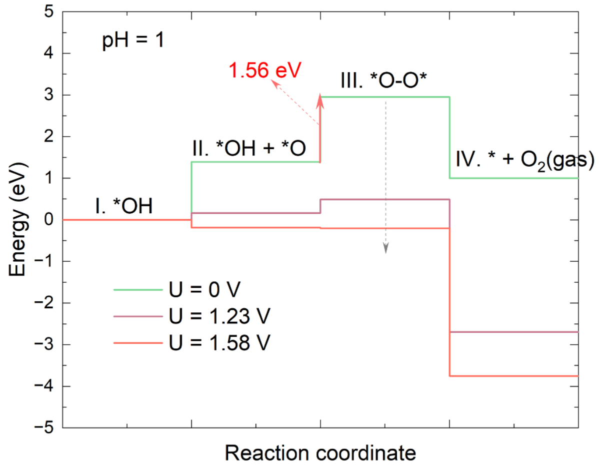

Supplementary Figure 19. DFT results of the OPM pathway

DFT calculations of the energy diagram for the OPM pathway in pH = 1, calculated with different electrode potentials (U = 0, 1.23 and 1.58 V). Source data are provided as a Source Data file.

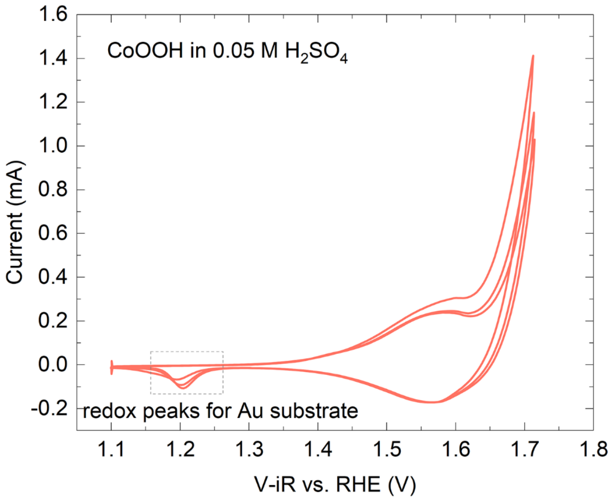

Supplementary Figure 20. CVs collected during operando measurement

The CVs (3 cycles) measurement in 0.05 M H_2SO_4 during operando XAS characterizations. The potential window is 1.1 ~ 1.72 V vs. RHE. The reduction peak at ~1.2 V vs. RHE caused by the Au layer coated on the electrode substrate for conductivity. Source data are provided as a Source Data file.

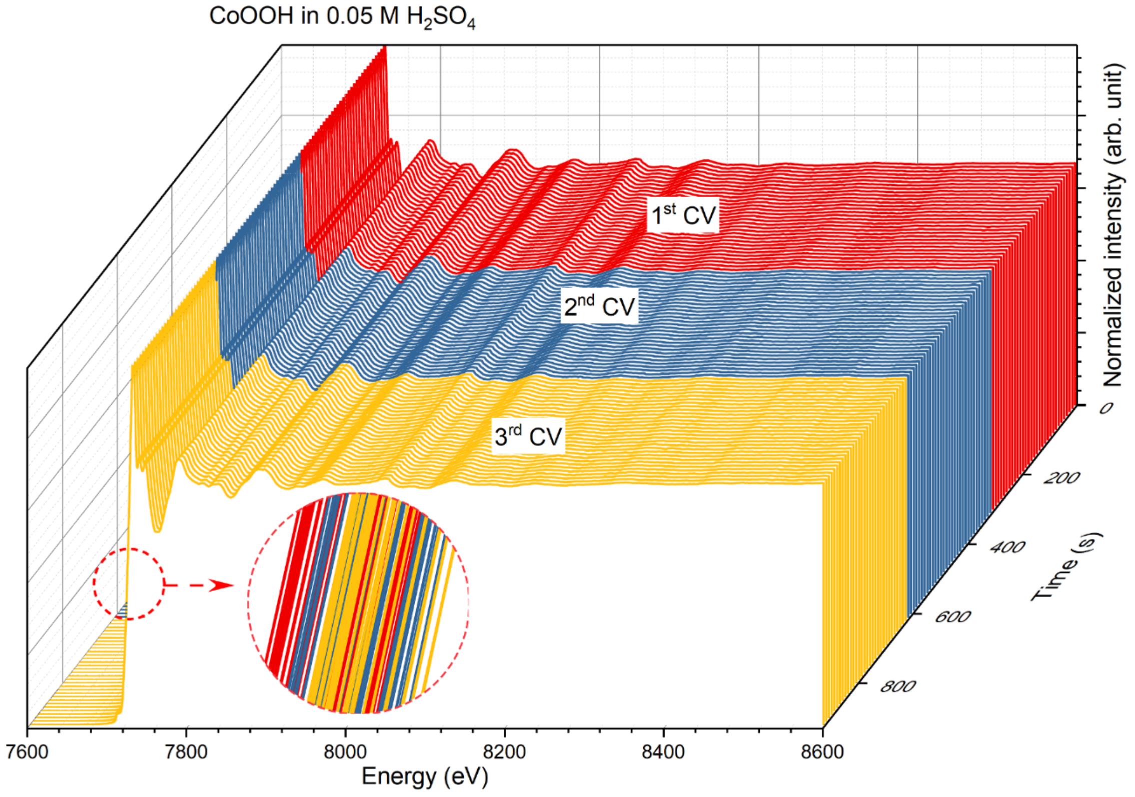

Supplementary Figure 21. Operando XAS spectra (CV)

Operando XAS spectra at the Co K edge of CoOOH during the 3 CV measurements in 0.05 M H_2SO_4. Every 20 spectra collected in 10 s were averaged into one to obtain better data quality. Source data are provided as a Source Data file.

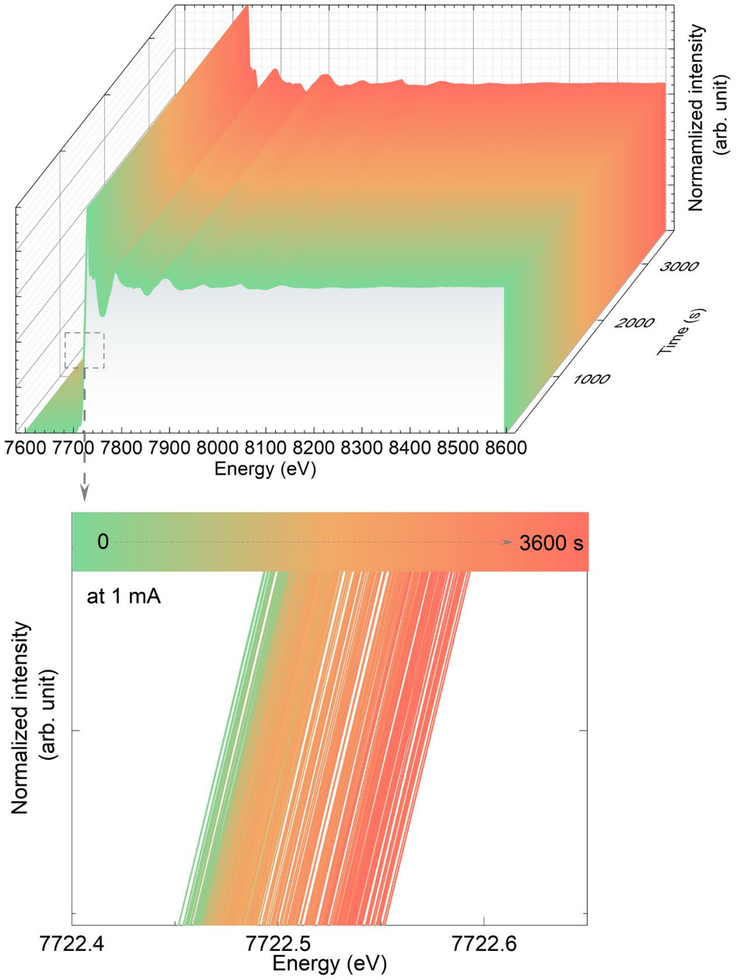

Supplementary Figure 22. Operando XAS spectra (CP)

Operando spectra at the Co K edge for CoOOH during the CP measurements in 0.05 M H_2SO_4 for 1h. Every 20 spectra collected in 10 s were averaged into one to obtain better data quality. Source data are provided as a Source Data file.

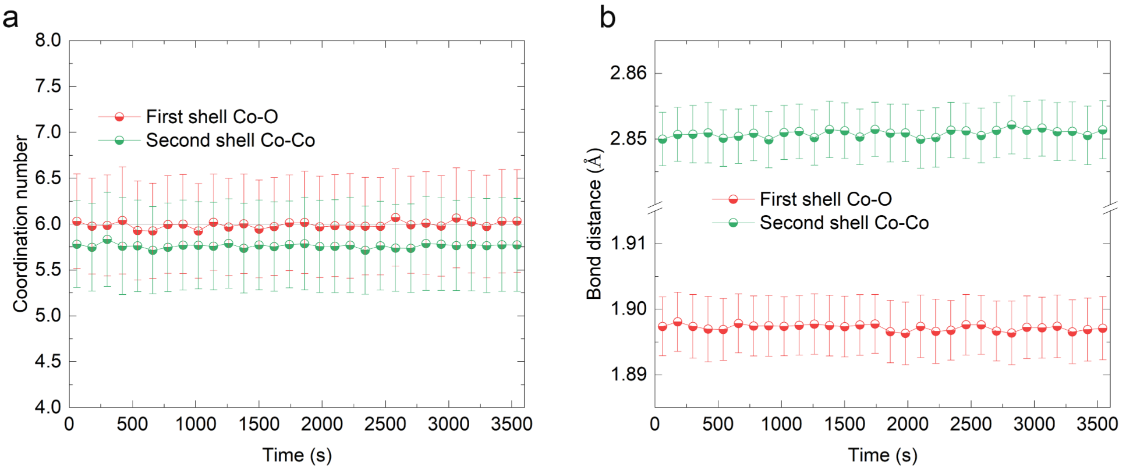

Supplementary Figure 23. Fitting of the operando EXAFS spectra (CP for 1 h)

(a) The coordination number (CN) and (b) the bond distance for the Co-O shell and Co-Co shell extracted from fitting the k^3-weighted EXAFS spectra in Figure 4d, which is plotted as a function of time. The error bar represents the error from the fitting in Artemis software. The fitting was performed at the k range of 2 to 14 $Å^{-1}$. Source data are provided as a Source Data file.

a b

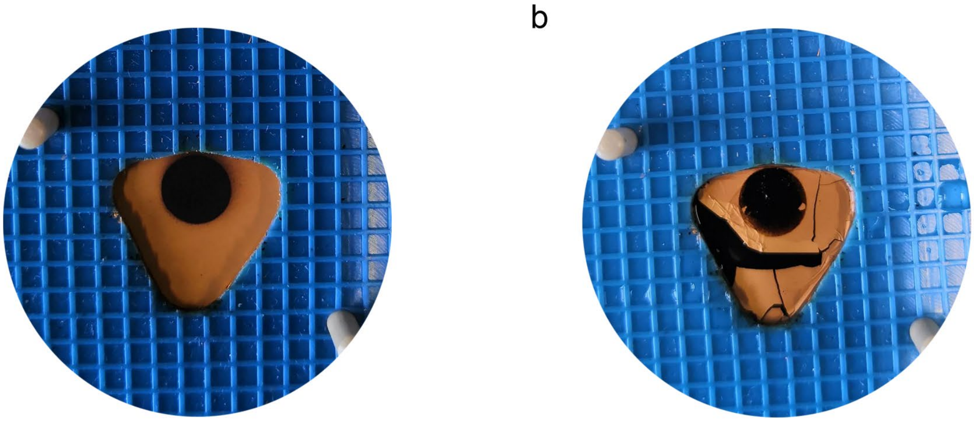

Supplementary Figure 24. Anode pictures of the flow cell

(a-b) Pictures of the anode electrode used in the flow cell (a) before and (b) after the CP test in an acidic electrolyte. The CoOOH was sprayed on the Au-coated Kapton foil. It is clear that the Au layer is delaminated, leading to the breakdown of the measured potential in Figure 5d. The CoOOH layer is still clearly observed on top of the Au layer. Source data are provided as a Source Data file.

Supplementary Table 5. Co dissolution

Summary of the Co dissolution during the CP measurement shown in Figure 5d and the corresponding stability number.

	Current (mA)	Duration (h)	Co dissolution (nmol)	Co dissolution rate (nmol h^{-1})	Stability number
CoOOH	1	43.2	576.9	13.3	699.3
CoOOH	0.5	56.9	380.1	6.7	698.3
Co_3O_4	1	24	305.4	12.7	732.8

Note: The stability number is calculated by $n(O_2)/n(Co)$ (ref.[12]), where n(Co) is the moles of dissolved Co detected in the electrolyte, and $n(O_2)$ is the moles of O_2 generated during CP measurement.

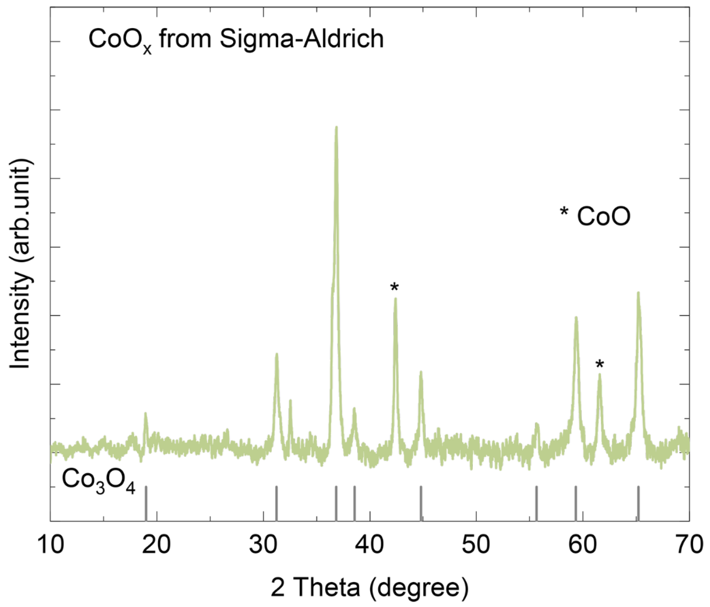

Supplementary Figure 25. Commercial CoO_x (Sigma-Aldrich)

XRD pattern of commercial CoO_x from Sigma-Aldrich, with Co_3O_4 as the main component and CoO as the secondary phase. Source data are provided as a Source Data file.

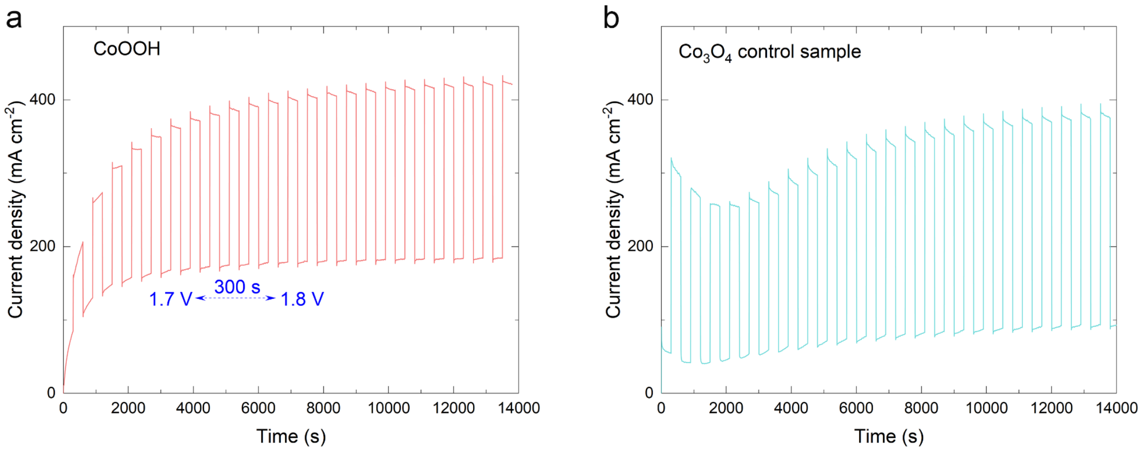

Supplementary Figure 26. Activation process

(a-b) The *i-t* curves of (a) the as-prepared CoOOH catalyst recorded during the conditioning process, where the cell potential was ramped between 1.7 V and 1.8 V every 300 s for ~ 45 cycles. (b) The Co_3O_4 control sample was also tested for comparison. We observe an activation process in both materials. Source data are provided as a Source Data file.

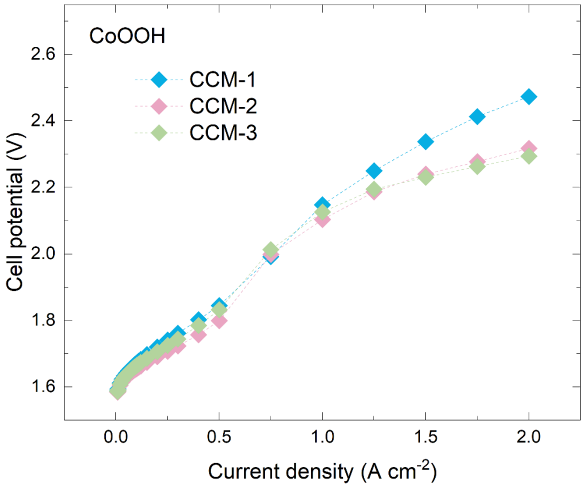

Supplementary Figure 27. Polarization curves of 3 CCMs

The polarization curves of three different CCMs with CoOOH as the anode catalyst. Source data are provided as a Source Data file.

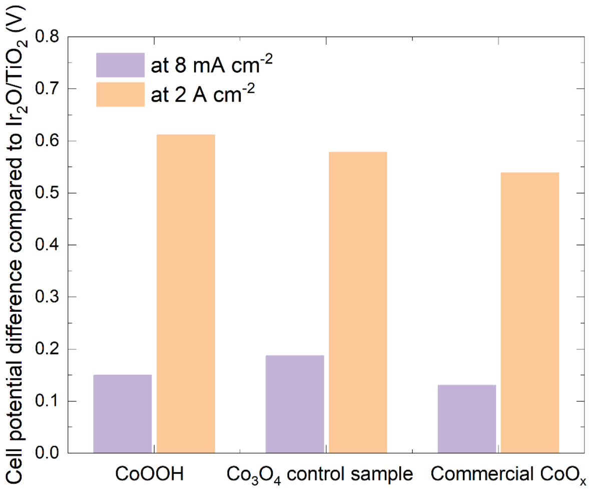

Supplementary Figure 28. Comparison of cell potential to IrO_2/TiO_2

The cell potential difference of Co-based catalysts (i.e., CoOOH, the Co_3O_4 control sample and commercial CoO_x) compared to the benchmark IrO_2/TiO_2 catalyst at a current density of 8 and 2000 mA cm^{-2}, respectively. Source data are provided as a Source Data file.

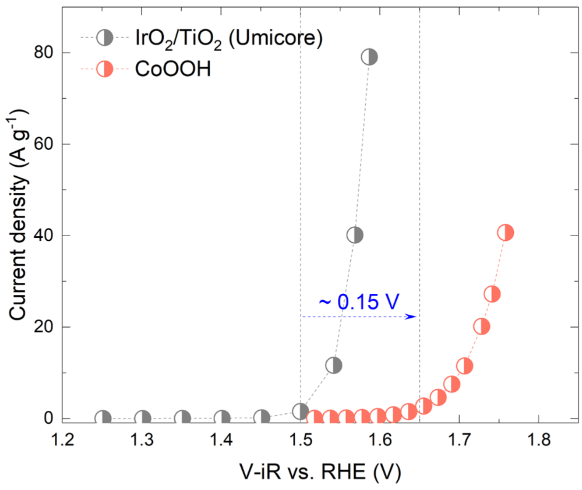

Supplementary Figure 29. Onset potential

Comparison of OER polarization curves of CoOOH and commercial IrO_2/TiO_2 in 0.05 M H_2SO_4, which were collected in a conventional three-electrode setup, with the catalyst dropcast on a rotating disk electrode. Obviously, the onset potential of CoOOH is ~ 0.15 V higher than that of IrO_2/TiO_2, consistent with the shift in the cell potential at low current densities in the PEM water electrolyzer. Source data are provided as a Source Data file.

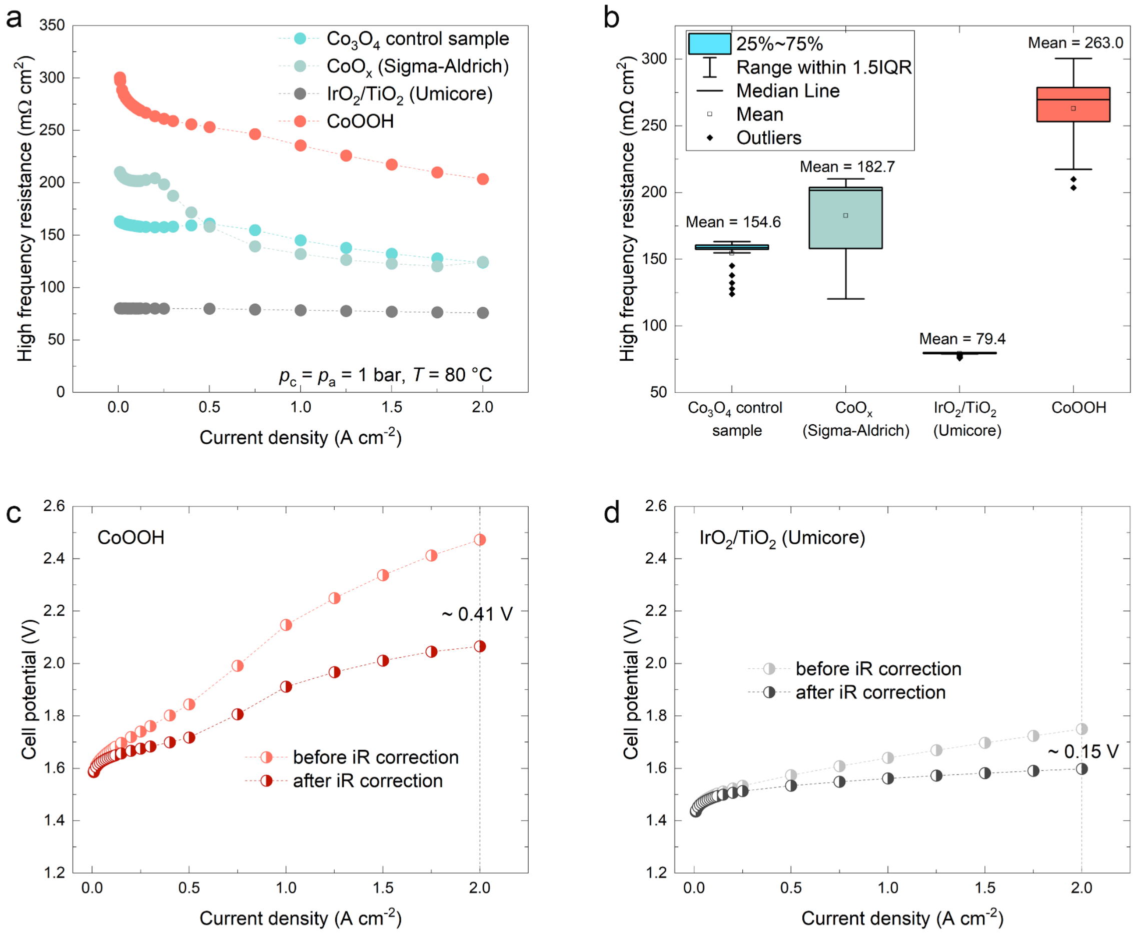

Supplementary Figure 30. HFR analysis

High frequency resistance (HFR) measured from the PEM water electrolysis cell. (a) The HFR of different catalysts is plotted as a function of the current density. (b) The box plot of the HFR to compare the mean HFR of different catalysts. (c-d) Comparison of the polarization curves before and after *iR* correction in (c) CoOOH and (d) IrO_2/TiO_2, respectively. Source data are provided as a Source Data file.

It can be clearly seen that the Co-based catalysts show higher HFRs compared to the benchmark IrO_2/TiO_2 catalyst. In addition, the HFR of IrO_2/TiO_2 is almost constant at different current densities, while the HFR of Co-based catalysts usually decrease with the current density.

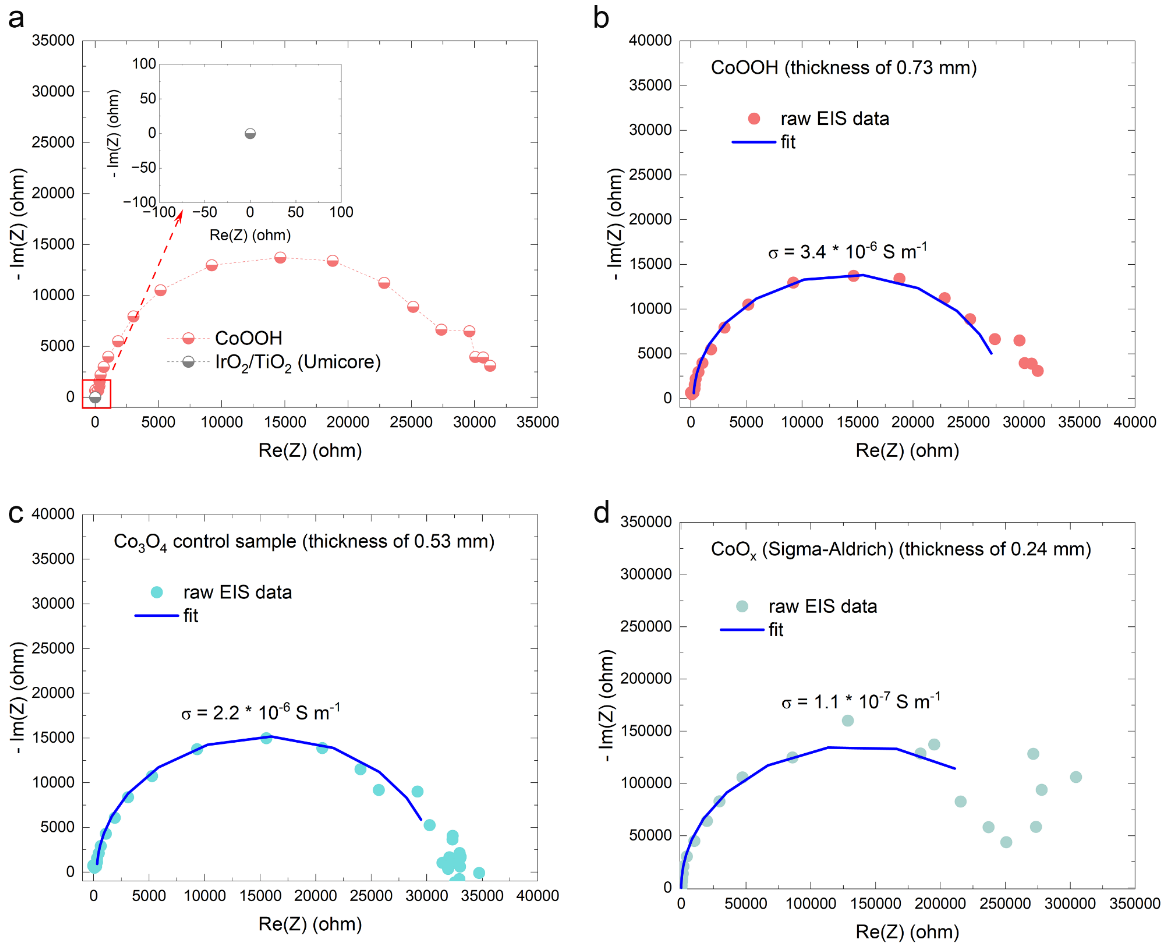

Supplementary Figure 31. Ex-situ conductivity

Ex-situ conductivity measurements on the catalyst powder using a four-electrode method, please check our previous papers[5, 13] for the details. The catalyst powder was pressed into a thin film for this measurement. (a) Comparison of the electrochemical impedance spectroscopy (EIS) spectra of semiconducting CoOOH with metallic IrO_2/TiO_2; the latter only shows a data point at ~ 0 ohm due to the short-cut occurring with good conductivity. (b-d) The raw EIS data (scatters) and the corresponding fit (blue curves) for (b) CoOOH, (c) Co_3O_4 control sample and (d) commercial CoO_x. Source data are provided as a Source Data file.

The raw EIS data was fitted with a (R1 + R2/C2) model to extract the resistance (R), then the conductivity (σ) of the catalyst powder is calculated by σ = l/(R*A), where l is the thickness of the catalyst, and A is the area of catalyst film. We note that for the Co-based catalysts studied here, the ex-situ conductivity follows the order of CoOOH > Co_3O_4 control sample > commercial CoO_x. The conductivity of the CoOOH and Co_3O_4 control samples studied here is also of the same magnitude as the CoO_x and CoO_x/CeO_2 catalysts that we prepared previously by flame spray synthesis[5].

Supplementary Note 2. Cell overpotential breakdown analysis.

We note that the cell potentials for all Co-based catalysts studied herein, i.e., the Co_3O_4 control sample, commercial CoO_x and CoOOH, increase very significantly at current densities above 500 mA cm^{-2}, resulting in a S-shaped polarization curve in Figure 6c[14, 15]. A similar phenomenon is also observed in the La and Mn co-doped Co_3O_4 catalyst (ref.[16]).

To better understand the different factors that contribute to the observed cell polarization curve, we performed the cell overpotential breakdown analysis Usually, the overpotentials can be categorized into three kinds: kinetic overpotential ($\eta_{kinetic}$), ohmic overpotential (η_{ohmic}) and rest overpotential (η_{rest})[17]. Specifically, the kinetic overpotential (Supplementary Figure 33a) is extrapolated from the fit of the linear part of the Tafel plots (Supplementary Figure 32a). The Tafel slopes determined for different catalysts are demonstrated in Supplementary Figure 33a. CoOOH shows a similar Tafel slope of 59.6 mV dec^{-1} to that of IrO_2/TiO_2 (55.1 mV dec^{-1}).

The ohmic overpotential is calculated from the current density and the corresponding HFR ($\eta_{ohmic} = i$ * HFR). Since the HFR of the Co-based catalysts is higher than that of IrO_2/TiO_2, the ohmic overpotential for Co-based catalyst is also higher (Supplementary Figure 33c). Moreover, we note that in Co-based catalysts, the rest overpotential contribution is as significant as the ohmic overpotential to the cell potential (Supplementary Figure 33d). It increases up to ~ 0.3 V at a current density of 2 A cm^{-2}. In comparison, the rest overpotential in IrO_2/TiO_2 is only ~ 0.037 V, close to the value seen in literature[17].

In addition, we examined the relationship between rest overpotential and current density. As the current density increases, the rest overpotential stays relatively low up to around 100 mA cm^{-2}, (Supplementary Figure 32b-e and Supplementary Figure 33b-d). It should be noted that at current densities of 30 mA cm^{-2} or below, the calculated rest overpotentials are subject to uncertainties arising from the limited validity of linear extrapolation of the kinetic overpotential in this region. When the current density is above 500 mA cm^{-2}, the rest overpotential rises sharply for both the Co_3O_4 control sample and for CoOOH. For CoO_x, this sharp increase in rest overpotential is observed at a comparably lower current density of 200 mA cm^{-2}. In literature, the rest overpotential is usually suggested to result from mass transport limitations or proton resistance in the CCM[14, 17]. Since, at a relatively high current density (for example, at 2 A cm^{-2}), the rest overpotential is significantly higher for the Co-based catalysts than for the benchmark IrO_2/TiO_2, with presumably similar catalyst layer structure, we speculate that mass transport does not constitute a major contributing factor in this case.

The proton resistance in the CCM can be responsible for the high rest overpotential. It is suggested that if there is cation contamination[14, 15] in a PEM water electrolyzer, the electric field can drive the contaminant cation to concentrate in the cathode, causing cathode proton depletion and thus increasing the local pH. At high current densities, the HER mechanism could change from proton reduction to water reduction due to the proton depletion[14].

Herein, we note that at low current densities the Co dissolution is relatively slow, then proton transfer from the anode to the cathode is still enough to support proton reduction. As the current density increases, the Co dissolution is faster, and the Co cation moves to the cathode via the membrane to cause proton depletion, and change the HER mechanism in the cathode from proton reduction to water reduction, resulting in the S-shaped polarization curves.

In the following Supplementary Note 3, we will compare the stability of CoOOH at current densities of 100 and 500 mA cm^{-2}, respectively, to show how the stability of CoOOH is related to the S-shaped polarization curves.

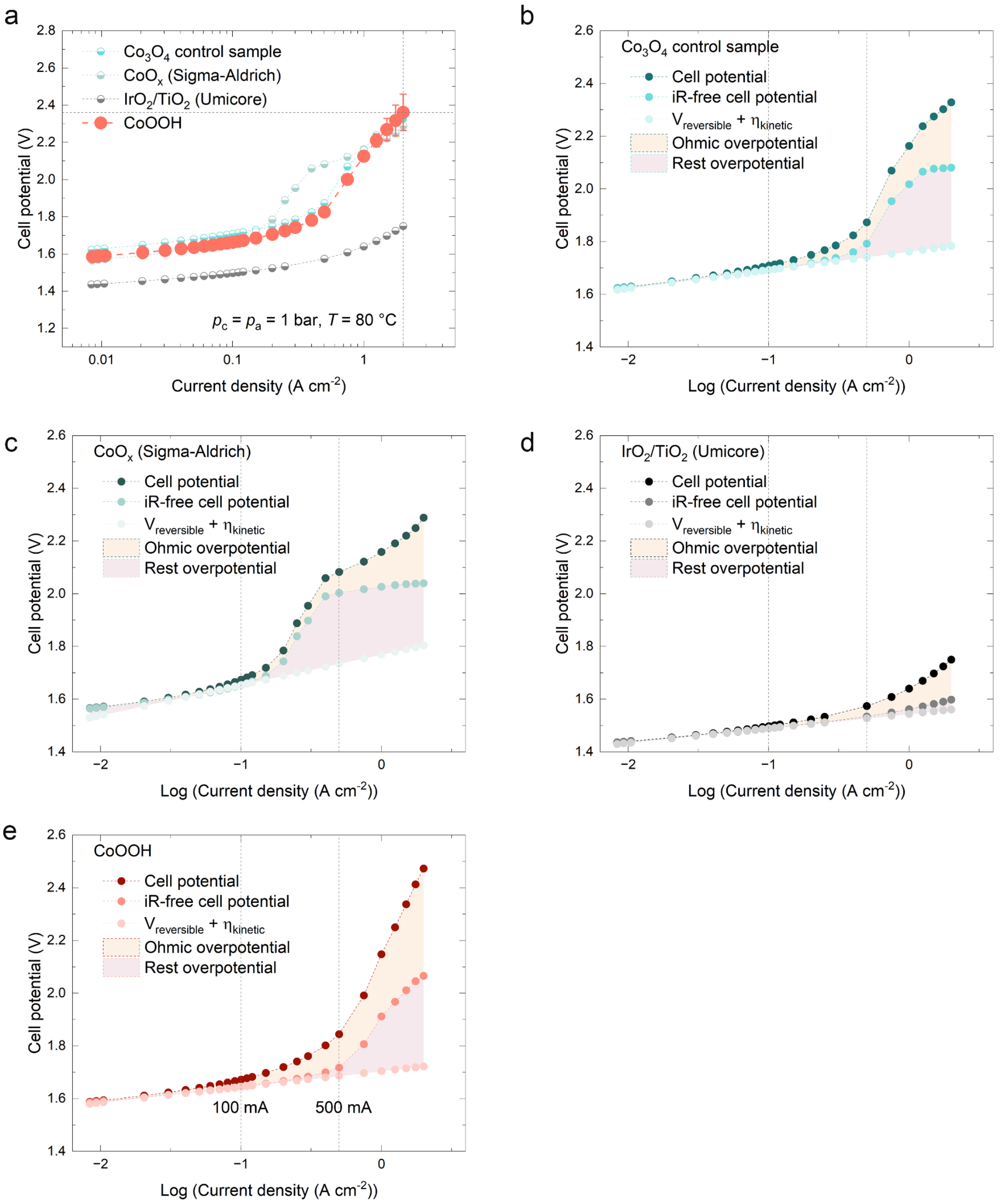

Supplementary Figure 32. Overpotential breakdown analysis

Overpotential breakdown analysis of polarization curves. (a) The Tafel plots, i.e., the polarization curves with a logarithmic scale of the current density for different catalysts. (b-d) The breakdown of the cell potential into reversible potential, kinetic overpotential, ohmic overpotential and rest overpotential for (b) the Co_3O_4 control sample, (c) commercial CoO_x, (d) CoOOH, and (e) IrO_2/TiO_2. The vertical dashed lines in panel (b-e) are located at current densities of 100 mA cm^{-2} and 500 mA cm^{-2}. Source data are provided as a Source Data file.

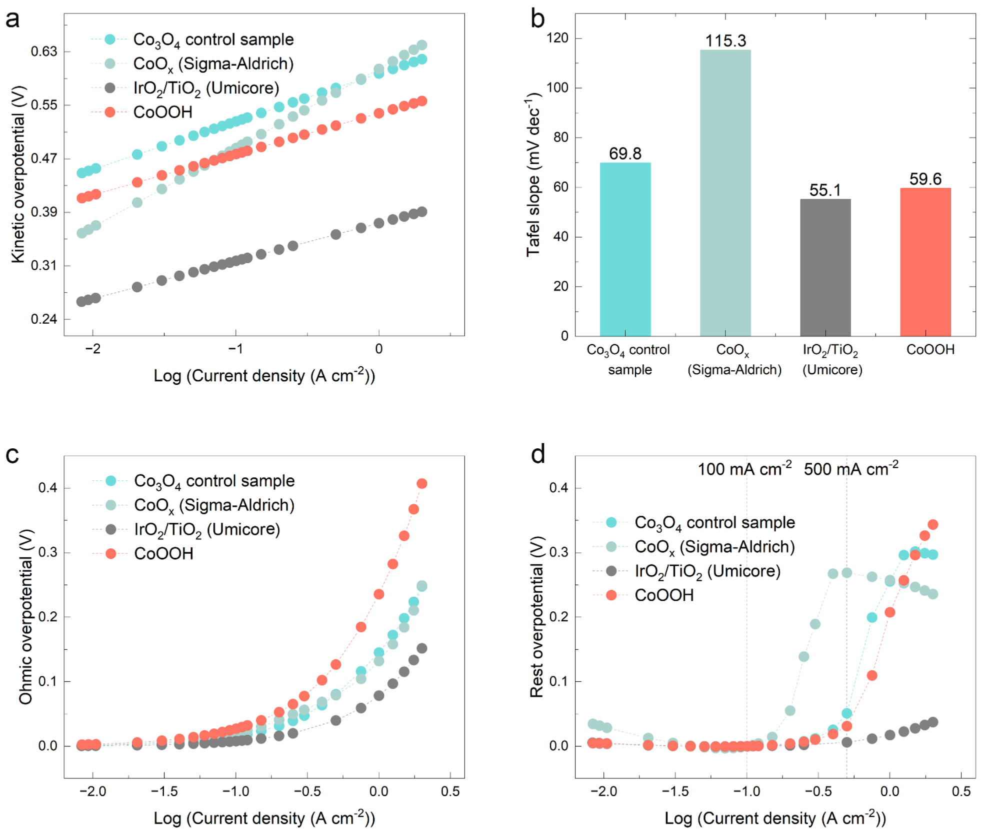

Supplementary Figure 33. Summary of overpotential breakdown analysis

(a-d) Summary and comparison of (a) the kinetic overpotential and (b) the corresponding Tafel slopes, as well as (c) the ohmic overpotential and (d) rest overpotential that are obtained from the overpotential breakdown analysis of polarization curves in Supplementary Figure 32. The vertical dashed lines in panel (d) are located at current density of 100 mA cm^{-2} and 500 mA cm^{-2}. Source data are provided as a Source Data file.

Supplementary Note 3. Stability of CoOOH in a PEM water electrolyzer.

As we point out in Supplementary Note 2, the S-shape polarization curve of CoOOH suggests that the rate of Co dissolution is different. The stability measurement was performed at current densities of 100 mA cm^{-2} (Figure 6c and Supplementary Figure 34-35), 200 mA cm^{-2} (Supplementary Figure 36) and 500 mA cm^{-2} (Supplementary Figure 37-38).

At a current density of 100 mA cm^{-2}, the rate of the water splitting reaction is mainly controlled by reaction kinetics, as confirmed by the linear Tafel plots in Supplementary Figure 35c-d. CoOOH shows good stability under these conditions, as revealed by the very stable CP curves in Figure 6c. Additionally, the cell potential in the polarization curve after a CP test even decreases due to a decreased rest overpotential. We propose that the surface of CoOOH can become more stable after conditioning at 100 mA cm^{-2} for 400 h, which reduces further Co dissolution and alleviates proton depletion at the cathode side, resulting in a smaller rest overpotential.

We note that the rest overpotential starts to increase obviously when the current density is above 200 mA cm^{-2}, due to the dissolved Co accumulating in the cathode and causing proton depletion. We show that the stability of CoOOH is indeed much worse at these conditions, as witnessed by an obvious of color change in the CCM after stability test at 500 mA cm^{-2} for 125 h (Supplementary Figure 37). The breakdown analysis of the polarization curves suggests the change in the cell potential is mainly due to a change in the kinetic overpotential, which can be explained by the significant decrease of the CoOOH loading on the CCM after Co dissolution.

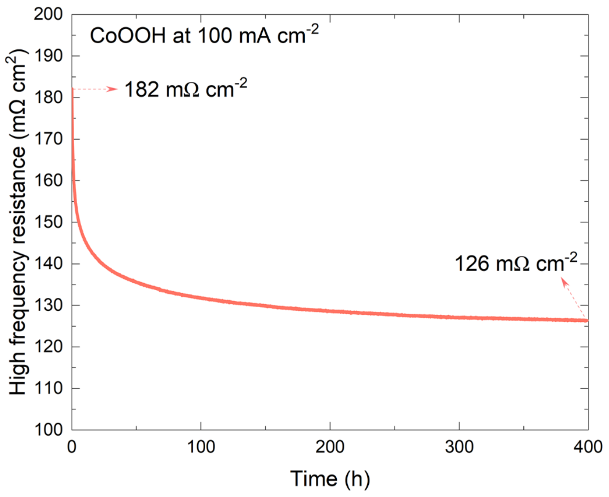

Supplementary Figure 34. HFR at 100 mA cm^{-2}

The HFR simultaneously measured during the CP at 100 mA cm^{-2} for 400 h. Source data are provided as a Source Data file.

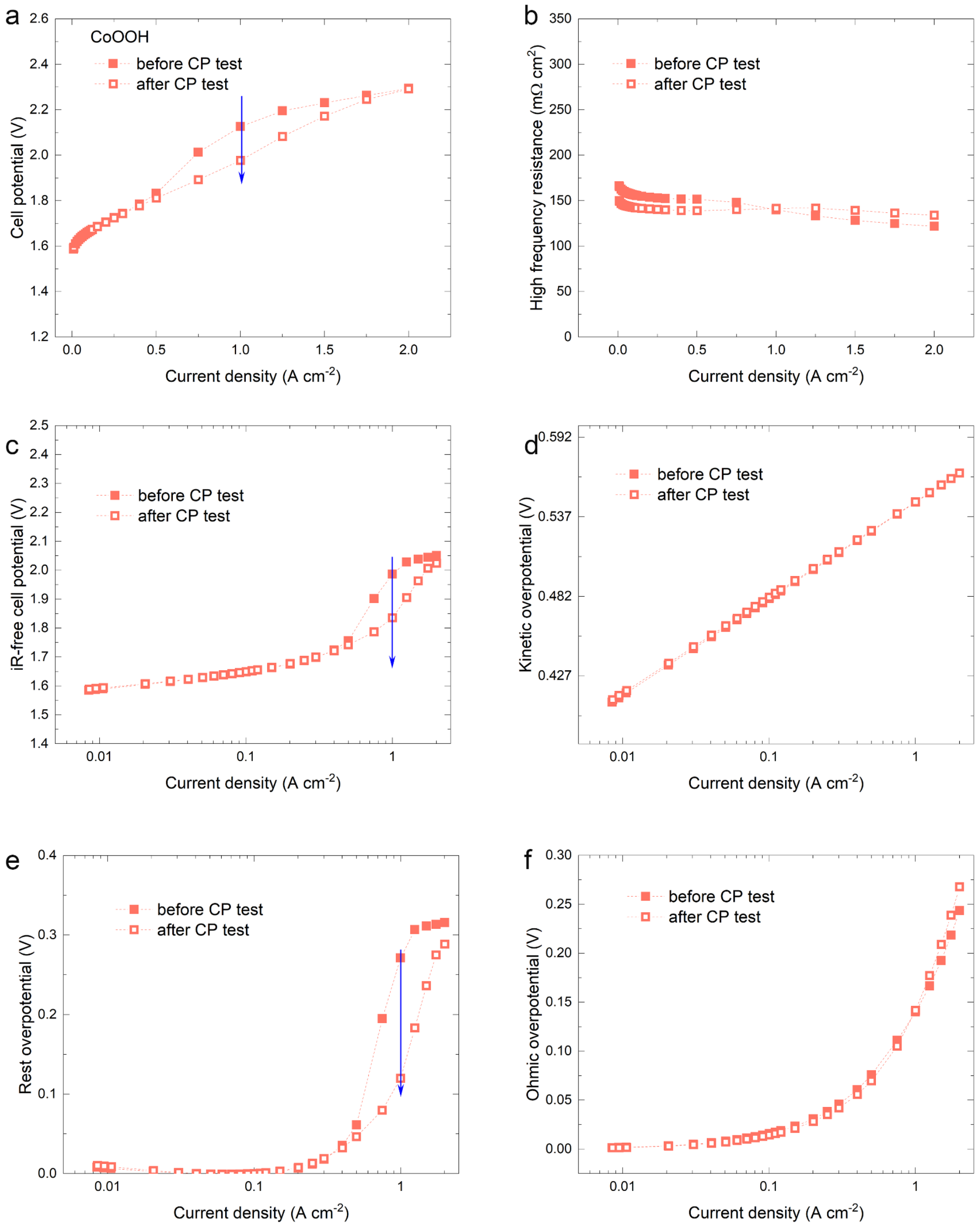

Supplementary Figure 35. Changes in overpotentials after CP at 100 mA cm^{-2}

Comparison and breakdown analysis of the polarization curves before and after the CP test at 100 mA cm^{-2} for 400 h. (a) The polarization curves and (b) the corresponding HFR before and after the CP test. (c) The Tafel plots and (d) the kinetic overpotential derived from the linear fit of the Tafel plots. (e) The rest overpotential and (d) ohmic overpotential extracted from polarization breakdown analysis. The vertical blue arrow indicates that the decrease in the cell potential after CP tests originates from a smaller rest overpotential. Source data are provided as a Source Data file.

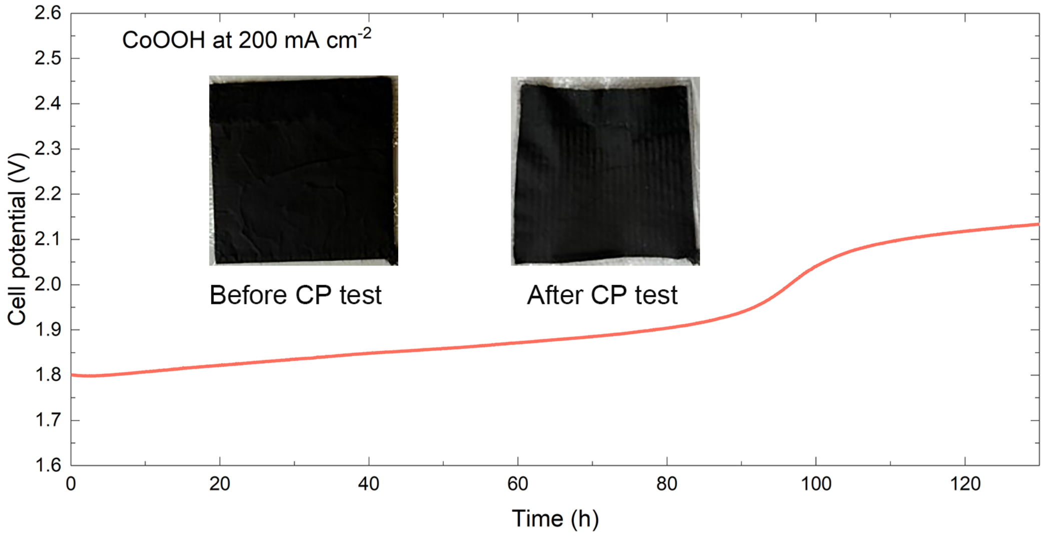

Supplementary Figure 36. CP measurement at 200 mA cm^{-2}

(a) The CP curve recorded at a constant current density of 200 mA cm^{-2} for 130 h. The inset pictures show the CCM at the CoOOH side before and after the CP test; a decrease in the darkness of the CCM indicates the dissolution of CoOOH under the condition of 200 mA cm^{-2}. Source data are provided as a Source Data file.

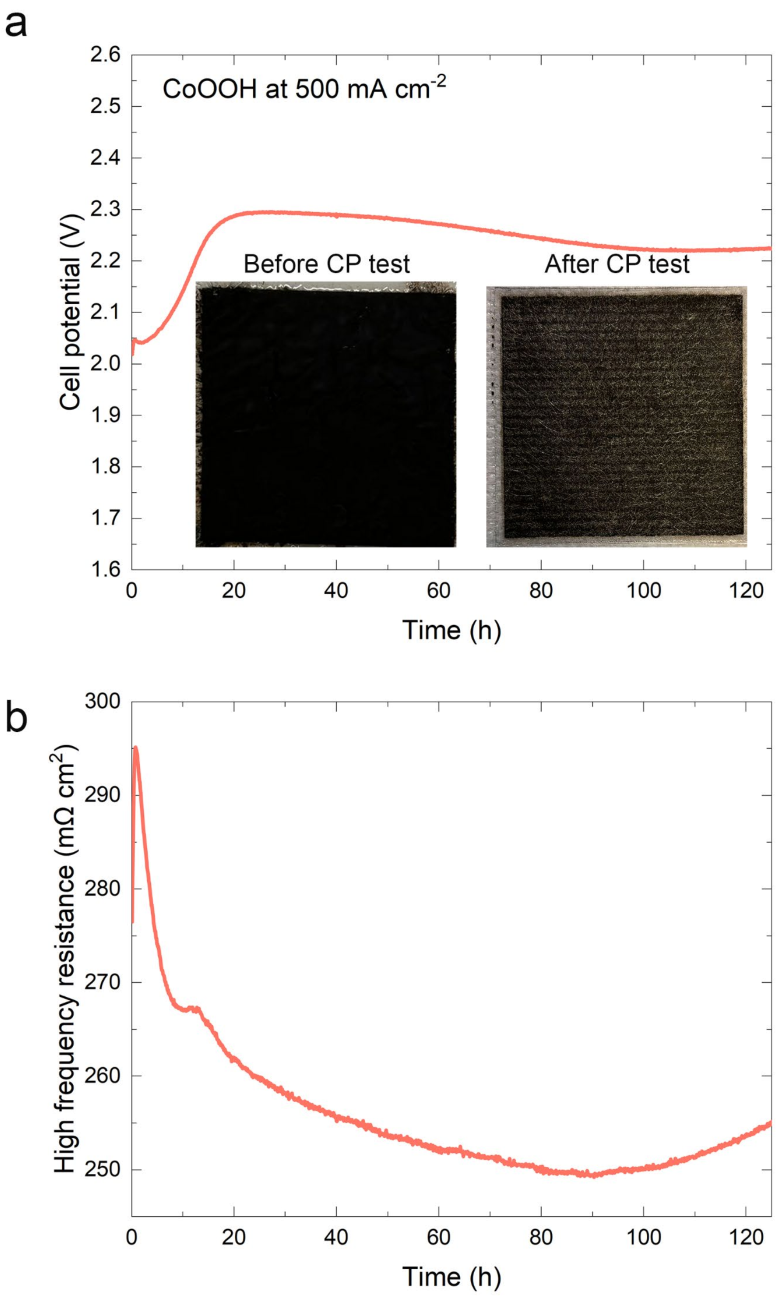

Supplementary Figure 37. CP measurement at 500 mA cm^{-2}

(a) The CP curve recorded at a constant current density of 500 mA cm^{-2} for 125 h. The inset pictures show the CCM at the CoOOH side before and after the CP test; a visible decrease in the darkness of the CCM indicates a significant dissolution of CoOOH under high current density. (b) The HFR measured simultaneously during the CP measurement. Source data are provided as a Source Data file.

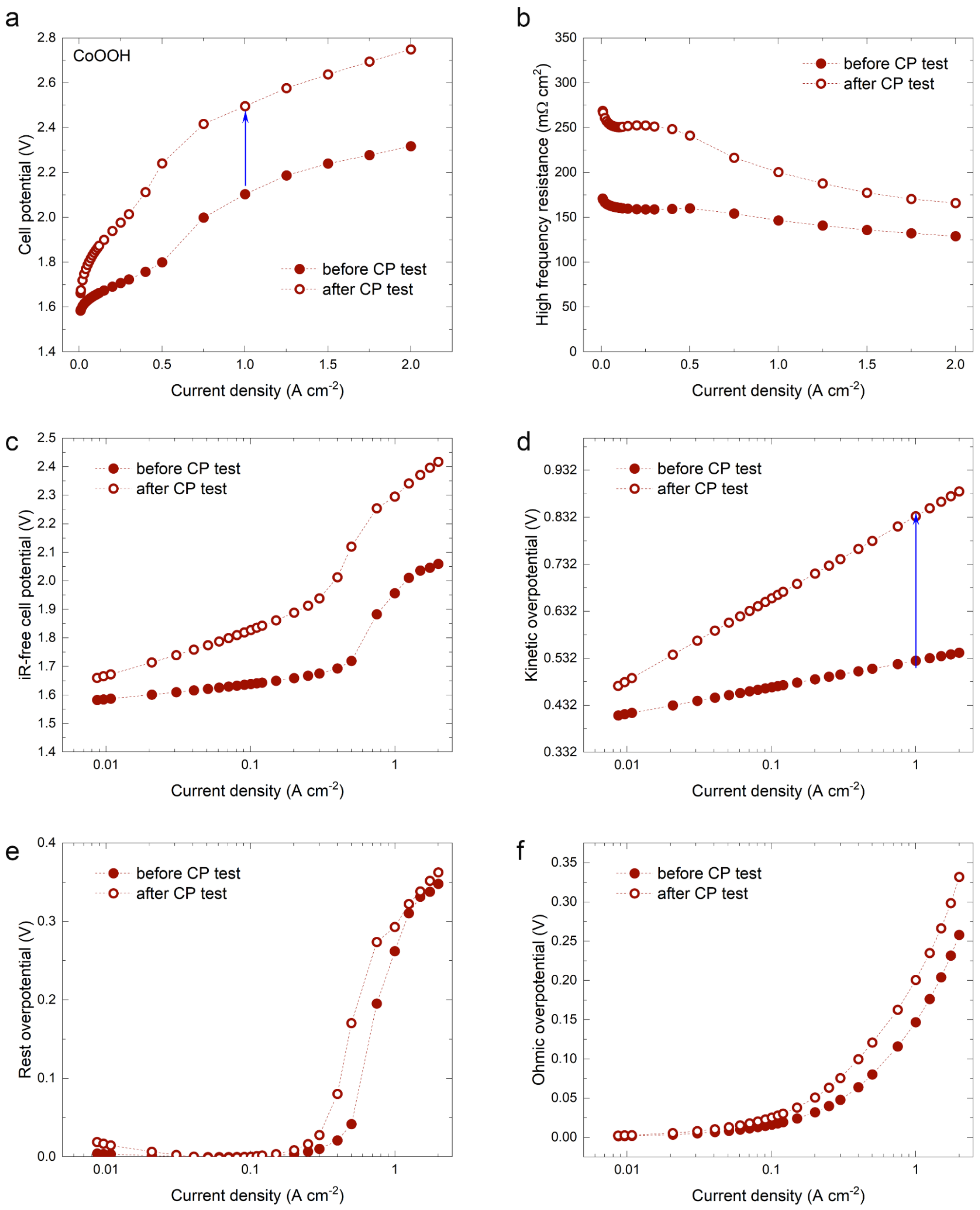

Supplementary Figure 38. Changes in overpotentials after CP at 500 mA cm^{-2}

A comparison and breakdown analysis of the polarization curves before and after the CP test at 500 mA cm^{-2} for 125 h. (a) Polarization curves and (b) the corresponding HFR before and after the CP test. (c) The Tafel plots and (d) the kinetic overpotential derived from the linear fit of the Tafel plots. (e) The rest overpotential and (d) ohmic overpotential extracted from overpotential breakdown analysis of polarization curves. The vertical blue arrow indicates that the decrease in cell potential after CP tests at 500 mA cm^{-2} for 125 h is mainly due to a change in the kinetic overpotential. Source data are provided as a Source Data file.

References in Supplementary information

1. Diercks JS, *et al.* Interplay between Surface-Adsorbed CO and Bulk Pd Hydride under CO2-Electroreduction Conditions. *ACS Catal.* **12**, 10727-10741 (2022).

2. Binninger T, *et al.* Electrochemical Flow-Cell Setup for In Situ X-ray Investigations. *J. Electrochem. Soc.* **163**, H906-H912 (2016).

3. Bott AW. Electrochemistry of semiconductors. *Curr. Sep.* **17**, 87-92 (1998).

4. Huang J, *et al.* Oxidation of interfacial cobalt controls the pH dependence of the oxygen evolution reaction. *Nat. Chem.* **17**, 856-864 (2025).

5. Huang JZ, *et al.* Operando Tracking the Interactions between CoO_x and CeO_2 during Oxygen Evolution Reaction. *Adv. Energy Mater.* **14**, 2303529 (2024).

6. Huang J, *et al.* Spectroscopic Investigations of Complex Electronic Interactions by Elemental Doping and Material Compositing of Cobalt Oxide for Enhanced Oxygen Evolution Reaction Activity. *Adv. Funct. Mater.* **34**, 2405384 (2024).

7. Ghoneim MM, Clouser S, Yeager E. Oxygen Reduction Kinetics in Deuterated Phosphoric Acid. *J. Electrochem. Soc.* **132**, 1160 (1985).

8. Sakaushi K. Observation of kinetic isotope effect in electrocatalysis with fully deuterated ultrapure electrolytes. *J.Electroanal. Chem.* **849**, 113372 (2019).

9. Lin Y, *et al.* Quantitative isotope measurements in heterogeneous photocatalysis and electrocatalysis. *Energy. Environ. Sci.* **13**, 2602-2617 (2020).

10. Gomez-Gallego M, Sierra MA. Kinetic isotope effects in the study of organometallic reaction mechanisms. *Chem. Rev.* **111**, 4857-4963 (2011).

11. Bajdich M, García-Mota M, Vojvodic A, Nørskov JK, Bell AT. Theoretical Investigation of the Activity of Cobalt Oxides for the Electrochemical Oxidation of Water. *J. Am. Chem. Soc.* **135**, 13521-13530 (2013).

12. Geiger S, *et al.* The stability number as a metric for electrocatalyst stability benchmarking. *Nat. Catal.* **1**, 508-515 (2018).

13. Cheng X, *et al.* Oxygen Evolution Reaction on Perovskites: A Multieffect Descriptor Study Combining Experimental and Theoretical Methods. *ACS Catal.* **8**, 9567-9578 (2018).

14. Padgett E, *et al.* Performance Losses and Current-Driven Recovery from Cation Contaminants in PEM Water Electrolysis. *J. Electrochem. Soc.* **171**, (2024).

15. Tan A, *et al.* Poisoning effects of Na-ions on membrane electrode assemblies in proton exchange membrane water electrolysis and strategies for recovery. *Chem. Eng. Sci.* **293**, (2024).

16. Chong L, *et al.* La- and Mn-doped cobalt spinel oxygen evolution catalyst for proton exchange membrane electrolysis. *Science* **380**, 609-616 (2023).

17. Garbe S, Futter J, Schmidt TJ, Gubler L. Insight into elevated temperature and thin membrane application for high efficiency in polymer electrolyte water electrolysis. *Electrochim. Acta* **377**, (2021).
