# Supplementary material for: Directly synthesized cobalt oxyhydroxide as an oxygen evolution catalyst in proton exchange membrane water electrolyzers
Source: Nat Commun. 2025 Aug 13;16:7518. doi: 10.1038/s41467-025-62744-4 (PMC12350611; doi:10.1038/s41467-025-62744-4)
Supplement: Supplementary file 2 — Description of Additional Supplementary Files [file 41467_2025_62744_MOESM2_ESM.pdf]

Description of Additional Supplementary Files

Supplementary Data 1

File Name: CoOOH_2O, CoOOH_O, CoOOH_OH, CoOOH_OO, CoOOH_OOH

Description: The CIF file showing the optimized intermediate structures on the (10-14) surface of CoOOH, and calculated results are shown in Figure 4d-e.

File Name: Energy_diagrams

Description: The jupyter notebook showing the details for DFT calculations.
